# Multi-omic analysis characterizes molecular susceptibility of receptors to SARS-CoV-2 spike protein

**DOI:** 10.1016/j.csbj.2023.11.012

**Published:** 2023-11-10

**Authors:** Fanjie Wu, Chenghao Lin, Yutong Han, Dingli Zhou, Kang Chen, Minglei Yang, Qinyuan Xiao, Haiyue Zhang, Weizhong Li

**Affiliations:** a Zhongshan School of Medicine, Sun Yat-sen University, Guangzhou 510080, China; b Key Laboratory of Tropical Disease Control of Ministry of Education, Sun Yat-Sen University, Guangzhou 510080, China; c Center for Precision Medicine, Sun Yat-sen University, Guangzhou 510080, China; d Department of Pathology, First Affiliated Hospital of Zhengzhou University, Zhengzhou 450052, China

**Keywords:** SARS-CoV-2 variant strain, Spike receptor, Binding affinity, Single-cell atlas, Transcriptomic analysis, BSG, Long COVID

## Abstract

In the post COVID-19 era, new SARS-CoV-2 variant strains may continue emerging and long COVID is poised to be another public health challenge. Deciphering the molecular susceptibility of receptors to SARS-CoV-2 spike protein is critical for understanding the immune responses in COVID-19 and the rationale of multi-organ injuries. Currently, such systematic exploration remains limited. Here, we conduct multi-omic analysis of protein binding affinities, transcriptomic expressions, and single-cell atlases to characterize the molecular susceptibility of receptors to SARS-CoV-2 spike protein. Initial affinity analysis explains the domination of delta and omicron variants and demonstrates the strongest affinities between BSG (CD147) receptor and most variants. Further transcriptomic data analysis on 4100 experimental samples and single-cell atlases of 1.4 million cells suggest the potential involvement of BSG in multi-organ injuries and long COVID, and explain the high prevalence of COVID-19 in elders as well as the different risks for patients with underlying diseases. Correlation analysis validated moderate associations between BSG and viral RNA abundance in multiple cell types. Moreover, similar patterns were observed in primates and validated in proteomic expressions. Overall, our findings implicate important therapeutic targets for the development of receptor-specific vaccines and drugs for COVID-19.

## Graphical Abstract

**Figure:**
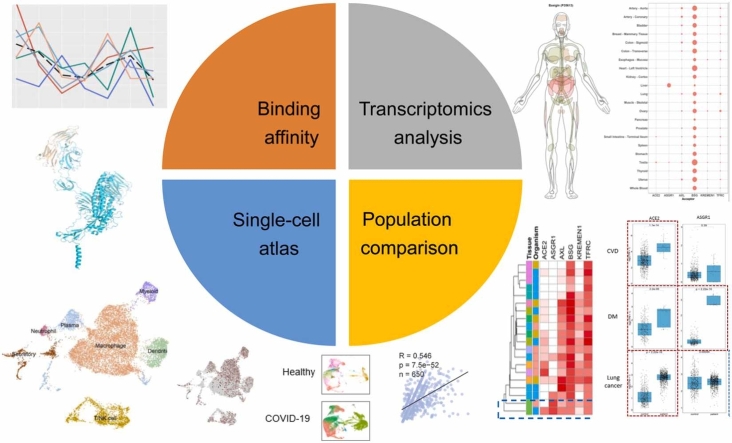

## 1 Introduction

The past coronavirus disease pandemic has caused more than 760 million COVID-19 cases and nearly 7 million deaths worldwide (www.who.int). In the post COVID-19 era, new variant strains may continue emerging [1], [2]. Compared with previous variants, the new variant acquired more immune escape capacity, which presented new epidemiological problems [3], [4], [5]. Meanwhile, long COVID is poised to be another public health challenge. A large cohort study [6] reported that SARS-CoV-2 infection is associated with 62 symptoms, and long COVID includes breathing and reproductive problems with a range of risk factors. Many of these symptoms might be related to multi-organ injuries and immune exhaustion [7]. Using multi-omic analysis including single-cell technologies can help to dissect the immunopathological signatures of COVID-19 [8], [9].

Beside the wild type of SARS-CoV-2 identified in the beginning of 2020 [10], six major variant strains have been officially reported by the World Health Organization (WHO). These variant strains were named as alpha (B.1.1.7), beta (B.1.351), delta (B1.617.2), gamma (P.1), lambda (C.37), and omicron (B.1.1.529) (Table 1). Among these, the omicron strain is currently the dominant variant worldwide and is continuing to mutate [11]. According to the WHO's classification, the lambda strain is considered a variant of interest (VOI), while the other strains are classified as variants of concern (VOC). Therefore, it is crucial to thoroughly and systematically investigate the molecular-level susceptibility of SARS-CoV-2 in order to address these emerging variants.

**Table 1:** Major SARS-CoV-2 variant strains and the structure information of their spike proteins.

	**Wild-type**	**Alpha variant**	**Beta variant**	**Delta variant**	**Gamma variant**	**Lambda variant**	**Omicron variant**
**Pango Name**	/	B.1.1.7	B.1.351	B.1.617.2	P.1	C.37	B.1.1.529
**WHO variant classification**	/	VOC	VOC	VOC	VOC	VOI	VOC
**Source database or method**	PDB	PDB	PDB	Homology Modeling	Homology Modeling	Homology Modeling	Homology Modeling
**Structure (template) ID**	6VYB	7N1X	7N1Q	7KRR.1. A (Template)	7KRR.1. A (Template)	7KRR.1. A (Template)	7N1V.1. A (Template)
**Key mutation sites (Sourced from GISAID)**	/	H69-, V70-, Y144-, N501Y, A570D, D614G, P681H, T716I, S982A, D1118H	D80A, D215G, L242-, A243-, L244-, K417N, E484K, N501Y, D614G, A701V	T19R, T95I, G412D, E156G, F157-, R158-, L452R, T478K, D614G, P681R, D950N	L18F, T20N, P26S, D138Y, R190S, K417T, E484K, N501Y, D614G, H655Y, T1027I, V1176	G75V, T76I, R246N, S247-, Y248S, L249-, T250-, P251-, G252-, D253-, S254-, L452Q, F490S, D614G, T859N	A67V, H69-, V70-, T95I, G142D, V143-, Y144-, Y145-, N211-, L212I, G339D, S371L, S373P, S375F, K417N, N440K, G446S, S477N, T478K, E484A, Q493R, G496S, Q498R, N501Y, Y505H, T547K, D614G, H655Y, N679K, P681H, N764K, D796Y, N856K, Q954H, N969K, L981F

GISAID: https://gisaid.org/

Footnotes：

Link to the outbreak.info database for mutation prevalence in the lineage

Wild-type, YP_009724390.1; Gisaid: EPI_ISL_402124 (see “clade evolution in the first year” on https://www.gisaid.org/);

Alpha variant: B.1.1.7-United-Kingdom; https://outbreak.info/compare/lineages?pango=B.1.1.7&gene=S&threshold= 0.2;

Beta variant: B.1.351-South Africa;

https://outbreak.info/compare-lineages?pango=B.1.351&gene=S&threshold= 0.2;

Delta variant: B.1.617.1-India;

https://outbreak.info/compare-lineages?pango=B.1.617&gene=S&threshold= 0.2;

Gamma variant: P.1-Japan/Brazil;

https://outbreak.info/compare-lineages?pango=P.1&gene=S&threshold= 0.2;

Lambda variant: C.37;

https://outbreak.info/compare-lineages?pango=C.37&gene=S&threshold= 0.2;

Omicron variant: B.1.1.529;

https://outbreak.info/compare-lineages?pango=B.1.1.529&gene=S&threshold= 0.2.

The spike protein of SARS-CoV-2 consists of S1 and S2 subunits, and it interacts with the host cell receptor through its receptor-binding domain (RBD) [12], [13]. When the S1 subunit recognizes the receptor, an enzyme cleavage site called TMPRSS2 is activated, leading to protein activation [14]. Subsequently, the spike protein undergoes a conformational change, facilitating membrane fusion between the virus and the host cell mediated by the S2 subunit [15], [16]. Host factors such as LY6E and IFITM also play a role in this process [17], [18]. Previous studies have shown a strong structural similarity between ACE2 and ACE, THOP1, and NLN oligopeptides, which raised the question of whether ACE, THOP1, and NLN may be involved in interactions with the spike protein [2], [19]. However, some organs that are susceptible to SARS-CoV-2 infection, such as the bronchus, esophagus, and liver, have low levels of ACE2 expression [20]. Even in the lungs, where infection symptoms are common, healthy alveolar epithelial cells have low ACE2 expression [19]. These findings suggest that ACE2 is not the sole receptor for the spike protein of SARS-CoV-2, highlighting the importance of investigating other potential receptors. Recent studies have shown that BSG, ASGR1, KREMEN1, AXL, and TFRC are receptors for the SARS-CoV-2 spike protein (Table 2). BSG, also known as CD147, is a membrane protein belonging to the immunoglobulin superfamily [21]. It can interaction with cyclophilins (CyP) to regulate the chemotaxis of leukocytes [22], [23], which is vital for recruiting leukocytes in rheumatoid arthritis and inflammatory lung diseases [24], [25], [26]. Moreover, BSG can also engage with multiple ligands, including inflammatory mediators like lymphocyte function-associated antigen (LFA-1) and CD43, with their levels significantly rising during injury [27]. Experimental evidence suggests that the spike protein of SARS-CoV-2 can bind to BSG, facilitating viral invasion [28], [29]. However, their interaction may differ from the interaction between ACE2 and the spike protein. Some studies indicate that the interaction between the spike protein and CD147 may not be directly related to viral entry into host cells, and CD147 may play a role in acting as an auxiliary binding receptor for the virus [30]. ASGR1 and KREMEN1 are transmembrane proteins that have been found to directly mediate SARS-CoV-2 infection by binding to spike proteins [31]. TFRC is involved in erythropoiesis and neurodevelopment [32], and it has been demonstrated that TFRC can interact with the spike protein to facilitate virus entry [33]. AXL, a member of the receptor tyrosine kinase subfamily, can interact with the NTD region of the spike protein and promote SARS-CoV-2 infection in human lung epithelial cells [34].

**Table 2:** The receptors of the SARS-Cov-2 spike protein and the structure information of their receptors.

**Receptors**	**Full name**	**Other names**	**Source database**	**ID**
ACE2	Angiotensin Converting Enzyme 2	ACEH	PDB[38]	6M1D
ASGR1	Asialoglycoprotein Receptor 1	CLEC4H1, HL-1	PDB	1DV8
AXL	AXL Receptor Tyrosine Kinase	UFO, JTK11, Tyro7, ARK	AFSTD[96]	AF-P30530-F1
BSG	Basigin	CD147, EMMPRIN, TCSF	AFSTD	AF-P35613-F1
KREMEN1	Kringle Containing Transmembrane Protein 1	KRM1, ECTD13	PDB	5FWS
TFRC	Transferrin Receptor	P90, CD71, IMD46, TFR1	PDB	2NSU

Deciphering the molecular susceptibility of receptors to the SARS-CoV-2 spike protein is crucial for understanding immune responses and multi-organ injuries in the face of emerging variants and long COVID. La Porta and Zapperi [35] estimated the binding affinities of SARS-CoV-2 peptides using artificial neural networks. Nawijn et al. [36] and Triana et al. [37] discussed the relationship between ACE receptor expression and susceptibility to infection. Vincenzo Tragni et al. predicted the affinity of new strains and potential new receptors by modelling binding affinity [2]. Affinity for the study was calculated in crystallized protein complexes or identical protein complexes after site-calculated mutations in vitro. However, integrating information from multiple omics levels is necessary to systematically explore molecular susceptibility and establish a comprehensive understanding of the novel coronavirus variants and receptors. This approach ensures that evidence supports each other and provides strong assistance for interventions against COVID-19.

In this study, we aimed to discover the molecular susceptibility of major receptors of SARS-CoV-2 spike protein by analyzing their binding affinities and their transcriptomic expression patterns, and expected to draw clinical attentions to be paid in tackling the infection and damage of different organs and tissues. We computed the binding affinities between major variant strains and different receptors of SARS-CoV-2 spike protein, and analyzed public transcriptomic data of 4100 experimental samples (including 3515 human samples for bulk RNA-seq, 276 for human single-cell RNA-seq, and 319 from primate bulk RNA-seq), and generated single-cell atlas based on more than 1.4 million cells across multiple tissues and organs of healthy and patients of COVID-19 and underlying diseases.

## 2 Results

### 2.1 Molecular docking and binding affinities between receptors and spike protein variants

**Fig. 1:**
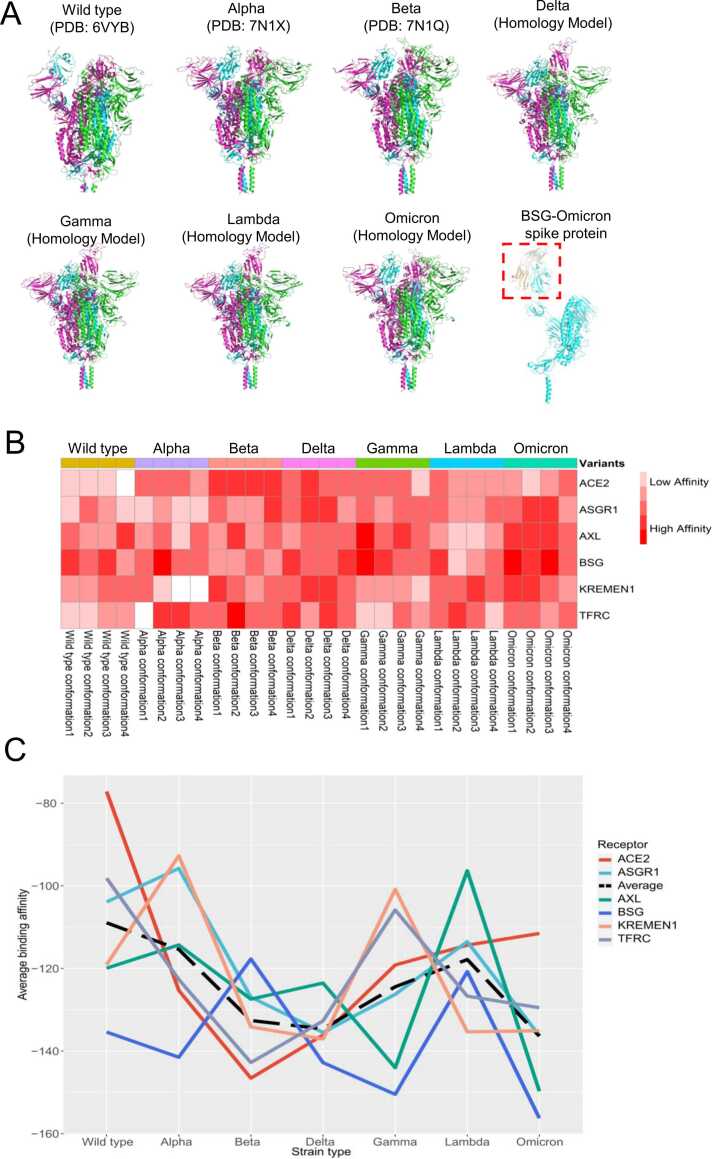
**The binding affinity analysis between human receptors and SARS-CoV-2 variant strains. (A)** The optimal structural conformations of the spike proteins for the seven SARS-CoV-2 variants, as well as the best binding conformations between the BSG receptor and the spike protein chains of the variant strains, are as follows. Each variant's spike protein consists of three chains. The trimeric spike protein structures of the wild type, alpha, and beta variants were obtained from crystallized proteins in the PDB database, with the three monomers adopting a mixed conformation (one up, two down). The spike protein structures of the remaining four variants were obtained through homology modelling, and the monomers also adopted the same mixed conformation. In the binding conformation of omicron-BSG, the BSG receptor is represented in yellow, one chain of the omicron variant spike protein is represented in light blue, and the binding site is indicated by a red rectangle with dashed lines. **(B)** The heatmap shows the binding affinity. Darker red color indicates stronger affinity. **(C)** The lines show the trends of affinity changes for the receptors against different variant strains.

Molecular docking simulations were performed to predict the protein binding affinities between the spike protein of SARS-CoV-2 and six receptors (ACE2, ASGR1, AXL, BSG, KREMEN1, and TFRC) in order to study the impact of mutations on the virus's binding capabilities. The structures of the variant strains and human receptors were obtained from the PDB database [38], the AlphaFold Protein Structure Database (https://alphafold.ebi.ac.uk), or through homology modeling using SWISS-MODEL (http://swissmodel.expasy.org/) [39] (Tables 1 & 2). The best structural conformations of the spike proteins for seven variants, as well as the best binding conformation between the BSG receptor and omicron's spike protein chain, are shown in Fig. 1A. The predicted binding affinities, based on full-length sequences and molecular docking, were clustered into a heatmap (Fig. 1B). The heatmap reveals that ACE2 exhibited a strong affinity with the SARS-CoV-2 beta strain, while ASGR1 showed affinity with the delta and omicron strains. AXL and BSG displayed affinity with the gamma and omicron strains, KREMEN1 with the delta and lambda strains, and TFRC with the alpha and beta strains.

To analyze the affinity trend among variant strains, we plotted trend lines (Fig. 1C) showing the average predicted affinity against the emerging strains from wild type to omicron. In Fig. 1C, the predicted binding affinity values are negative, and larger absolute values indicate stronger affinities. The trend lines reveal that the delta strain exhibited strong predicted affinities with all receptors. The wild type and alpha strains had the strongest predicted affinities with BSG, beta strain with ACE2 and TFRC, lambda strain with KREMEN1, and gamma and omicron strains with BSG and AXL. The average trend line indicates that omicron had the strongest predicted affinity with the receptors compared to other strains, with delta being the second strongest. This partly explains why omicron is currently the predominant strain worldwide, while delta was the major strain previously. Interestingly, the trend also shows that BSG had the highest predicted binding affinities with the wild type, alpha, delta, gamma, and omicron strains. Even though the structure of the spike-CD147 protein complex has not been experimentally determined.

### 2.2 Transcriptomic expression profiles of human receptors at bulk RNA-seq level

**Fig. 2:**
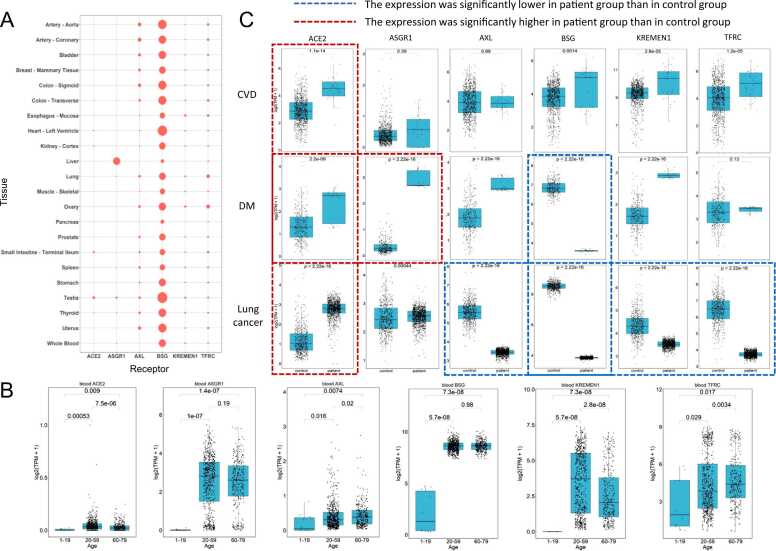
**The transcriptomic analysis at tissue level. (A)** The bubble diagram illustrates the transcriptomic expression levels of 6 receptors across 22 human tissues/organs. **(B)** Comparative analysis of transcriptomic expression among different age groups. **(C)** Comparative analysis of transcriptomic expression among different underlying disease states (CVD, MD, and lung cancer).

To investigate the susceptibility of various human tissues to SARS-CoV-2, the median expression matrix data for the 6 receptors from 2312 human samples were collected from the GTEx database and a bubble diagram was drawn based on the expression data clustering (Fig. 2A). The expression pattern indicates that at least one of the six receptors has a high-level expression in any of 21 human tissues. Surprisingly, ACE2 showed significantly low expressions in most tissues compared to other receptors, while BSG was highly expressed across all the 21 tissues, especially in testis and heart. Interestingly, ASGR1 was highly expressed in liver only.

To explore the different susceptibility between children, adults, and elders, Wilcoxon test was performed to compare the RNA expressions of the receptors in whole blood samples between three age groups (10 children of 1–19 years old, 607 adults of 20–59 years old, and 322 elders of 60–79 years old). We found that the median expression levels of the six receptors were significantly lower in children than in adults and elders. Moreover, the BSG expression in children concentrated at a much lower level than that in adults and elders (Fig. 2B).

Several diseases, including cardiovascular disease (CVD), diabetes mellitus (DM), and lung cancers, have been proven to increase the risk of COVID-19 infection [40], [41]. Among them, CVD and DM are two prevalent underlying diseases with a large number of patients, while lung cancer has the highest incidence rate among respiratory system cancers. We examined the expression of six receptors in these underlying disease states by comparing the expression profiles of healthy samples from GTEx (861 heart, 328 pancreas, and 578 lung tissues) with patient samples from The Cancer Genome Atlas (TCGA) database (1129 lung cancer) and GEO databse (34 CVD and 30 DM). ACE2 expression was significantly higher in all three patient populations compared to controls (Fig. 2C, 1st column). ASGR1 showed higher differential expression in DM patients compared to CVD and lung cancer (Fig. 2C, 2nd column). Surprisingly, BSG expression was significantly lower in DM and lung cancer patients compared to controls (Fig. 2C, 4th column). In lung cancer patients, the average expression of AXL, BSG, KEEMEN1, and TFRC was very low compared to controls (Fig. 2C).

### 2.3 Transcriptomic expression profile at bulk-RNA-seq level in primates

**Fig. 3:**
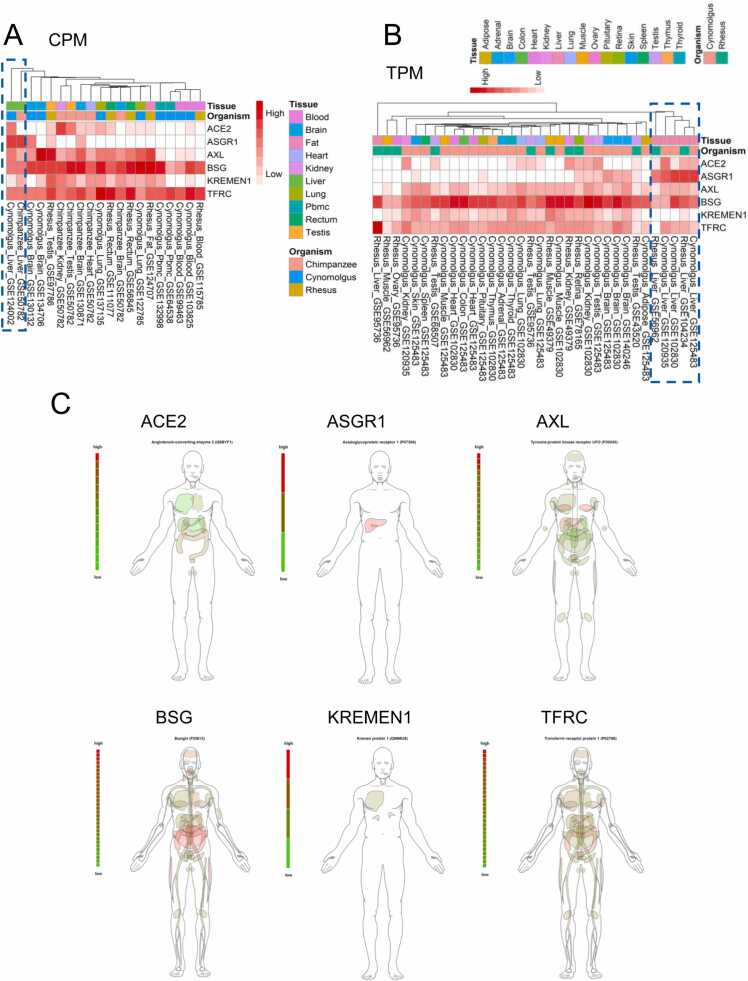
**The transcriptomic expression in primates and the proteomics expression in human. (A)** The transcriptomic expression heatmap displays the expression levels of various receptors in different tissues/organs of three primate animals, represented by CPM values. **(B)** The transcriptomic expression heatmap illustrates the expression levels of different receptors in various tissues/organs of three primate animals, using TPM values. **(C)** The proteomic expression heatmap showcases the expression of different receptors in human tissues/organs.

To explore the susceptibility of SARS-CoV-2 in various tissues of other primates, including rhesus monkey (*Macaca mulatta*), cynomolgus monkey (*Macaca fascicularis*), and chimpanzee (*Pan troglodytes*), we analyzed the transcriptomic expression data of 319 primate samples collected from the Gene Expression Omnibus (GEO) database. We generated two expression heatmaps (Fig. 3A&B) using the source datasets of two quantification types - Transcripts Per Kilobase per Million mapped reads (TPM) and Counts Per Million mapped reads (CPM) [42]. The heatmaps show the clustering of expression patterns of the six receptors across different tissues of the three primates. According to the two heatmaps, ACE2 was lowly expressed in most tissues of three primates, and only highly expressed in kidney, liver, and testis of rhesus monkey and cynomolgus monkey (Fig. 3A&B) and in kidney and testis of chimpanzee (Fig. 3A). BSG was highly expressed across most tissues of primates. It is also notable that the three primates had very similar expression patterns of receptors in liver (blue dash boxes), such as high expression of ASGR1 and low expression of KREMEN1, very similar to the expression patterns in human liver.

### 2.4 Receptor expression patterns on single-cell atlases in healthy people

**Fig. 4:**
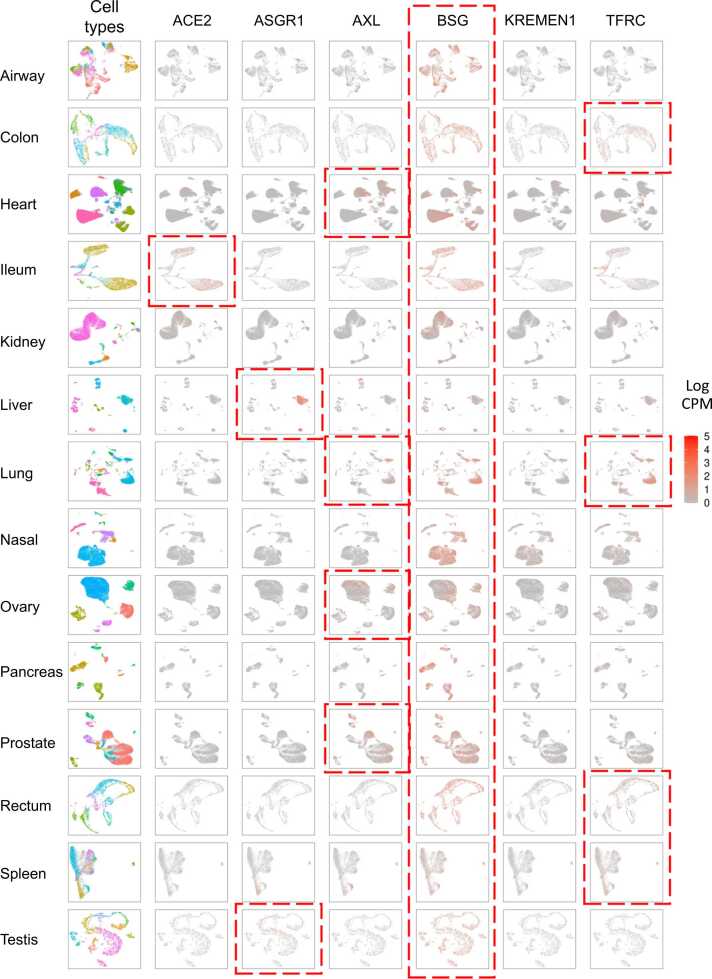
**The transcriptomic analysis for healthy individuals at single-cell level.** The rows correspond to various tissues/organs, while the first column indicates the cell types present in those tissues/organs. The remaining columns represent the expression levels of each receptor in different tissues/organs. The scale has been normalized using log CPM.

To explore the susceptibility of various cells in human tissues at a single-cell level, we collected single-cell transcriptomic data of healthy samples from the UCSC Cell Browser to draw the single-cell atlases and the expression scatter plots of receptors (Fig. 4). The single-cell data included 810,350 cells from 163 healthy samples across 17 organs or tissues. We found BSG was widely and highly expressed across all the tissues compared to other receptors, while KREMEN1 was rarely expressed across all the tissues. ACE2 was only highly expressed in ileum and kidney. AXL was highly expressed in heart, lung, ovary, and prostate. TFRC had high expressions in colon, lung, rectum, and spleen.

**Fig. 5:**
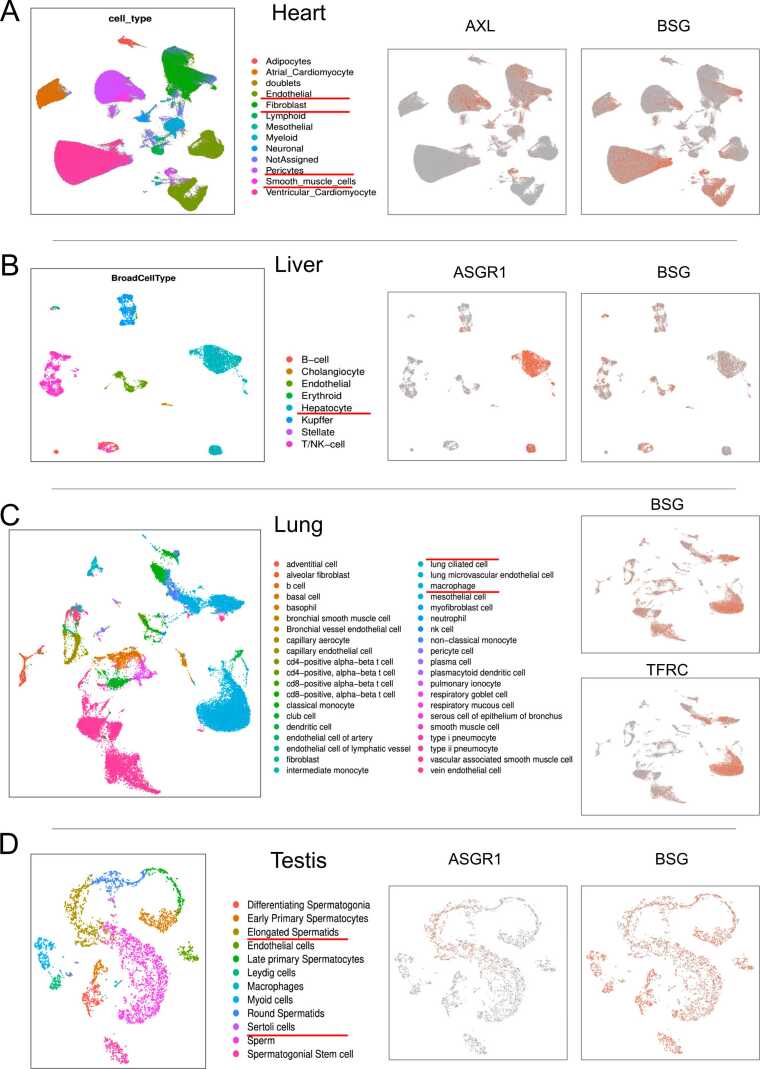
The single cell atlases for heart, liver, lung, and testis of healthy human, and specific receptors’ expression patterns on the atlases.

In the respiratory tract, the nasal mucosa and airway were the first body parts to come into contact with external air. Interestingly, only BSG showed high expression in all cell types of these two body parts compared to other receptors. This pattern of high BSG expression was also observed in the colon, ileum, lung, pancreas, prostate, rectum, and testis. In the heart, AXL was highly expressed in fibroblast and pericytes (Fig. 5A), while BSG was highly expressed in endothelial and smooth muscle cells. The liver showed high expression of BSG, particularly in the hepatocyte cells (Fig. 5B). In the lung, most cell types exhibited high expression of BSG, while macrophages and mesothelial cells also highly expressed TFRC (Fig. 5C). BSG was the only receptor widely expressed in the testis, with high expression observed throughout the process of spermatogenesis (Fig. 5D). Elongated spermatids and Sertoli cells in the testis also highly expressed ASGR1 (Fig. 5D).

### 2.5 Receptor expression patterns on single-cell atlases between healthy and COVID-19 patients

**Fig. 6:**
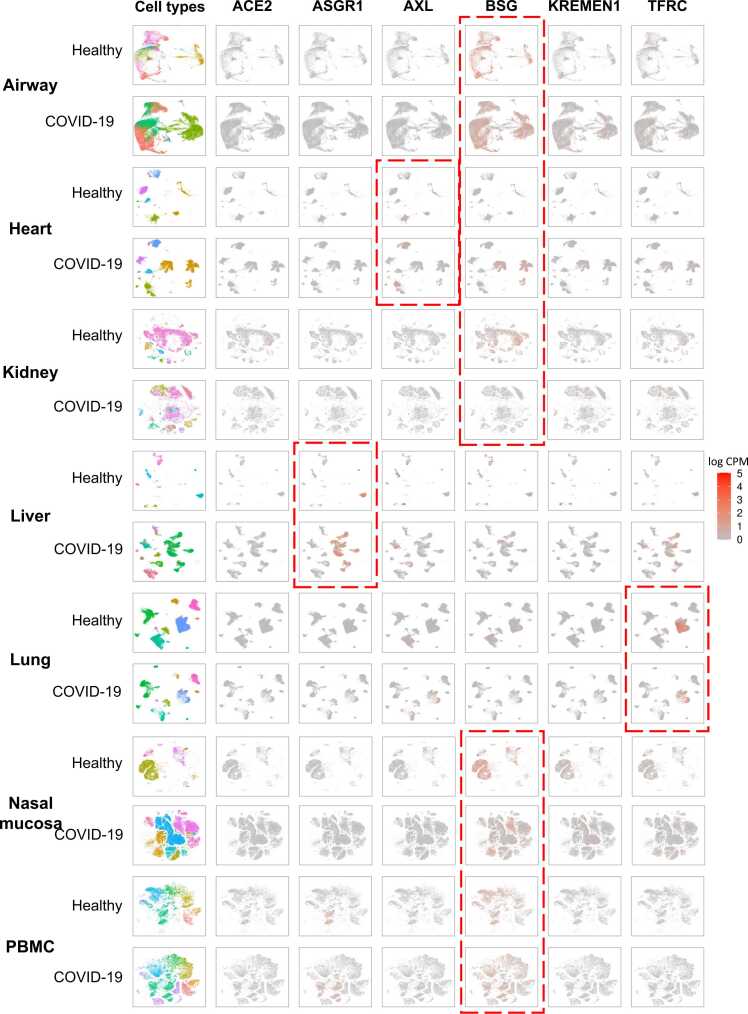
**The transcriptomic comparison between healthy and patients at single-cell level.** The rows represent different tissues/organs in healthy or patient samples, while the first column displays the cell types corresponding to these tissues/organs. The remaining columns indicate the expression levels of each receptor in the different tissues/organs of healthy or patient samples. The expression levels have been normalized using log CPM.

Transcriptomic single-cell sequencing data of 810,350 cells from 163 normal samples and 402,295 cells from 90 COVID-19 patient samples across airway, heart, kidney, liver, lung, nasal mucosa, and PBMC (peripheral blood mononuclear cell) were downloaded from UCSC Cell Browser [43] or GEO database and analyzed using general single cell analysis methods (see Methods). UMAP [44] and t-SNE [45] were utilized to cluster and plot the cell types and the expression patterns (Fig. 6). Consistent with the transcriptomic result aforementioned, ACE2 had low expressions and shows no much difference between healthy and patient samples across 7 organs (airway, heart, kidney, liver, lung, nasal mucosa, and PBMC). BSG had high expressions in both healthy and patients across airway, nasal mucosa, and PBMC samples.

**Fig. 7:**
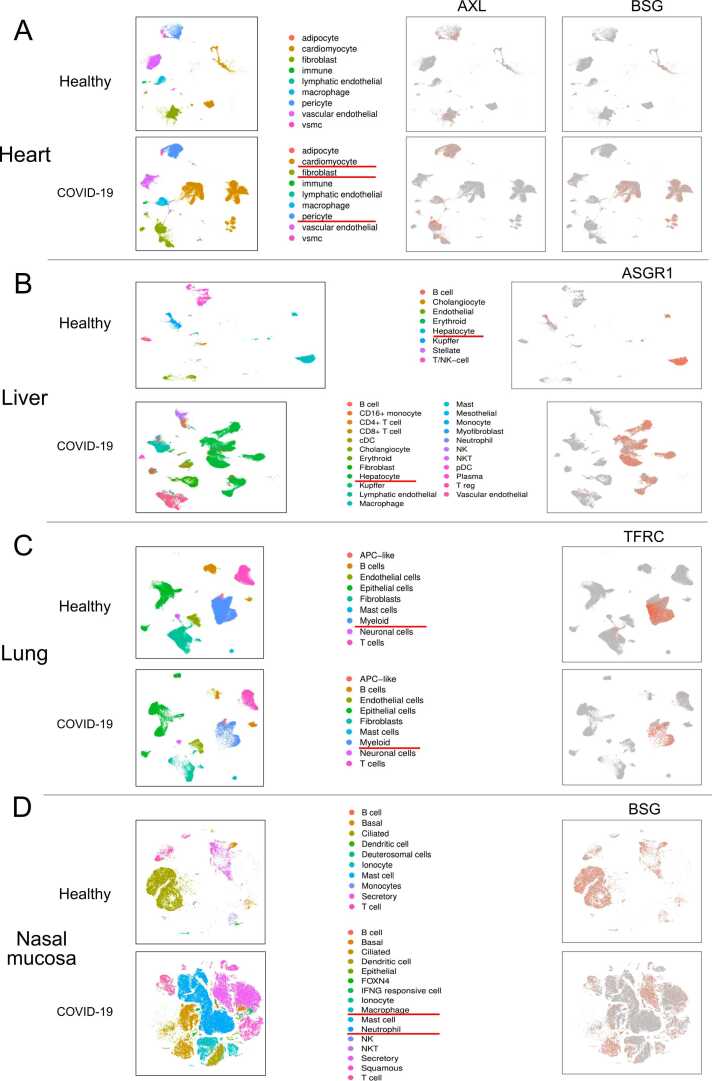
The single cell atlases for heart, liver, lung, and nasal mucosa between COVID-19 patients and healthy controls, and specific receptors’ expression patterns on the atlases.

COVID-19 patients showed increased cell types in the airway, heart, liver, and nasal mucosa compared to healthy controls. In the heart, patients had more cell types and higher expressions of AXL and BSG in specific cell types (Fig. 7A). Kidney samples showed little difference in cell types but lower BSG expression compared to healthy samples. Liver samples from patients had more cell types and high ASGR1 expressions in hepatocyte cells (Fig. 7B). Lung samples had similar cell types between healthy and patients, with TFRC showing high expressions in myeloid cells (Fig. 7C). Nasal mucosa samples from patients had more cell types, such as neutrophil cells and macrophages (Fig. 7D). PBMC samples showed similar cell types and expression patterns of the 6 receptors between healthy and patients (Fig. 6). Overall, COVID-19 affected the microenvironments and showed distinct expression patterns of certain receptors in different organs.

### 2.6 Correlation analysis between receptor expression and viral infection at a single-cell level

In order to investigate the relationship between virus infection and the presence of specific receptors at the single-cell level, we analyzed additional single-cell transcriptome sequencing data from COVID-19 patients. These datasets, obtained from the GEO database (GSE145926 and GSE155249), contained a total of 222,968 cells, including over 10 thousand SARS-CoV-2-RNA-positive cells.

**Fig. 8:**
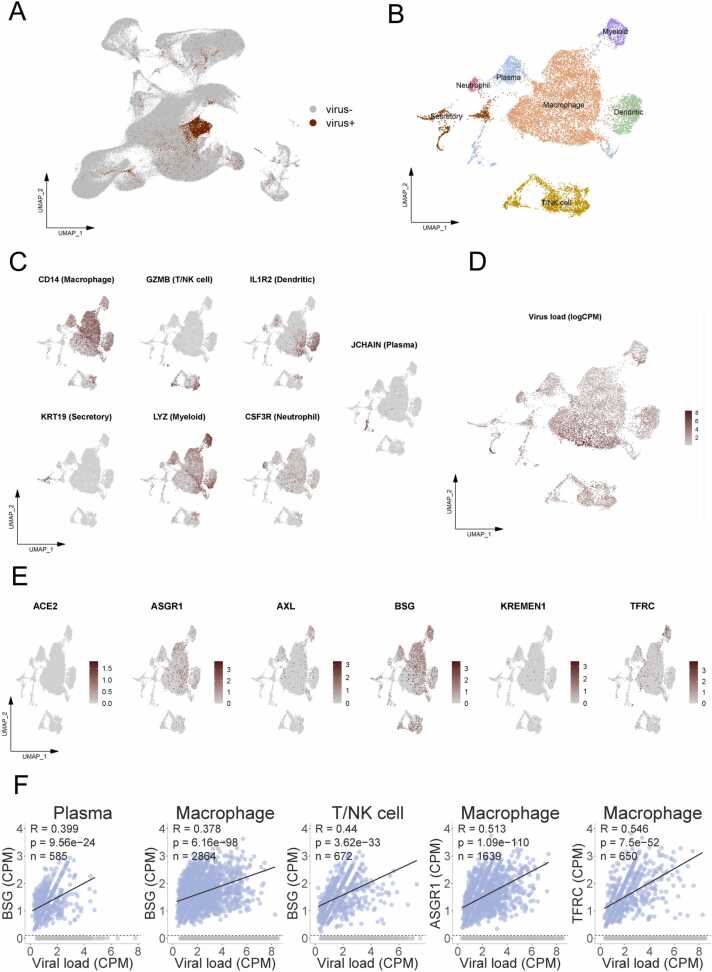
**Cell types with SARS-CoV-2 RNA detected and specific receptors’ expression patterns in SARS-CoV-2 RNA-positive cells.** (A) 13,916 SARS-CoV-2-RNA-positive cells were detected (UMI > 0) in the datasets of GSE145926 and GSE155249. (B) UMAP of the cell types of SARS-CoV-2 RNA-positive cells. (C) The marker genes used to determine cell types. (D) Viral load in SARS-CoV-2 RNA-positive cells quantified by log(CPM). (E) Expression levels of specific receptors in SARS-CoV-2-RNA-positive cells. (F) Pearson’s correlations of BSG, ASGR1 and TFRC’s expression patterns in different cell types (R>0.3, n > 500, p < 0.05) with viral load (CPM) (zero-expression cells were excluded from regression analysis to reduce the effects of dropouts).

As the comparison process used a customized reference genome that added the SARS-CoV-2 genome as an extra chromosome to the human genome, it was possible to detect both viral RNA and human gene expression at the single-cell level. After a unified process, we detected viral RNAs of SARS-CoV-2 in 13,916 cells (Fig. 8A and B) from secretory cells and a diverse set of immune cells, including myeloid cells, neutrophils, macrophages, plasma cells, dendritic cells, and T/NK cells. The identity of these SARS-CoV-2-RNA-positive cells was confirmed by the corresponding marker genes (Fig. 8C). Interestingly, these cells were more commonly found in immune cells than in epithelial cells (Fig. 8D). We also examined the expression levels of six receptors in these SARS-CoV-2 RNA-positive cells (Fig. 8E). BSG showed the highest expression level and was present in the largest number of cells. ASGR1, TFRC, AXL, and KREMEN1 also showed significant expression, while ACE2 expression was minimal. It is worth noting that ACE2 was not expressed in immune cells.

To ensure a reliable correlation analysis, we focused on receptors that were expressed in more than 500 SARS-CoV-2-RNA-positive cells in each cell type. We found that BSG was moderately associated with viral RNA abundance (R>0.3 and p < 0.01) in macrophages, plasma cells, and T/NK cells. Similarly, ASGR1 and TFRC showed similar correlations in macrophages (Fig. 8F). Other correlations between receptor’s expression (100 <n < 500) and viral RNA abundance are shown in Fig. S2.

### 2.7 Validation on proteomic expression level for human receptors

We tried to verify the results of human transcriptomic expression profile at the proteomics level. We searched the protein expressions of 6 receptors at the ProteomicsDB database and retrieved the visualizations (Fig. 3C) of the protein expression patterns, which are involved 586 human proteomic samples. We found that ACE2 in various tissues and organs showed low protein expressions, similar to that in transcriptomic data. ASGR1 illustrated specifically high expressions in liver at both proteomics and transcriptomics levels. Similarly, the protein expressions of AXL in breast and liver were significantly higher compared to other organs. Heart, colon, and testis had high expressions of BSG protein, which were also confirmed by the transcriptomic expressions as aforementioned. The protein expression of TFRC in digestive tract was significantly higher than that in other tissues. In summary, these receptors exhibited similar expression patterns at both transcriptomics and proteomics levels.

## 3 Discussion

### 3.1 Binding affinities between different receptors and variant spikes explain the domination of delta and omicron

Molecular docking predicts the binding strength and interaction mode between ligand and receptor molecules based on the lock and key principle. This method has been used to predict the binding affinity of various SARS-CoV-2 variants to ACE2 receptors [46], [47], [48], [49]. Other tools have also been developed and utilized, and their descriptions and comparisons can be found in the Methods section of this study. Furthermore, our calculations align with the real-world epidemic trends of different strains and tissue infections [28], [50].

The omicron and delta strains of SARS-CoV-2 show the first and second predicted strongest affinities with receptors compared to other strains. This is consistent with the fact that these strains have been dominant in the current and previous outbreaks. The analysis of protein predicted binding affinities between different receptors and variant spikes can provide insights into the susceptibility of SARS-CoV-2 at the molecular level.

It's worth emphasizing that, apart from ACE2, the interactions between ASGR1, KREMEN1, and TFRC (confirmed through co-immunoprecipitation assays) [31], [51], as well as AXL (confirmed through in vitro binding assays) [34], with the spike protein have been experimentally validated. In contrast, the binding of BSG to the spike protein remains speculative at present. Furthermore, the predicted affinities of BSG and AXL for the spike protein by the AlphaFold model are solely based on modelling predictions, and the structure of the spike protein-CD147 protein complex has not been definitively established yet. The flexibility and conformational changes of the spike protein are crucial for successful infection [52], [53]. These conformational changes, which involve the opening or closing of the three RBDs of the spike protein, determine its ability to bind to receptors [54]. The different combinations of RBD conformations result in varying affinities for receptor binding [4]. These factors limit the accuracy of our binding affinity calculations.

Recently published studies by molecular experiments supported our results of the strain affinities for ACE2. Ramanathans’s study on the interaction between the RBD region and ACE2 using microscale thermophoresis showed that the binding affinities of beta and alpha strains to ACE2 were 4.62 and 1.98 times to that of wild-type [47], other studies have found similar results [55]. Barton et al. analyzed the surface plasmon resonance and found that most mutations in RBD of alpha, beta, and gamma trains increased the affinity between RBD and ACE2 [46]. Molecular experiments based on an enzyme linked immunosorbent assay (ELISA) showed that omicron strain had weaker binding affinity to human ACE2 than delta strain [56]. Biolayer interferometry showed that gamma and beta strains similarly increased the affinity to ACE2 compared to wild type [57]. Besides these, the spike protein of delta strain showed a moderate increase in ACE2 affinity compared with wild-type, which has also been demonstrated by experiment in previous studies [58].

### 3.2 High expression patterns suggest the importance of BSG for multi-organ injuries and long-COVID

Among the 6 receptors, ACE2 has lower expressions in human organs at the tissue transcriptomics, single-cell transcriptomics, and proteomics levels, as well as in primates. This lower expression is observed in both healthy control and COVID-19 patient samples across multiple human organs. On the other hand, BSG is highly expressed in most human organs and in primates. BSG also has high expressions in both healthy controls and COVID-19 patients in airway, nasal mucosa, and PBMC samples.

Multiple studies have shown low ACE2 expressions but high BSG expressions in various tissues and cell lines. For example, Aguiar et al. found that ACE2 protein expression was rarely observed in airway epithelial cells, while BSG had extensive expression in respiratory mucosa [59]. Qiao et al. demonstrated low ACE2 expression and high BSG expression in human brain cell lines using quantitative real-time polymerase chain reactions (qRT-PCR) and Western blotting [60]. These findings suggest the existence of alternative receptors, such as BSG, for SARS-CoV-2 to infect host cells. However, unlike ACE2, which directly mediates viral infection, some studies suggest that the binding of BSG to the spike protein may not be directly associated with viral entry into host cells, and its role may be that of serving as an auxiliary binding receptor for the virus [30].

In our study, we found that the receptor BSG (Basigin) was widely and highly expressed across 14 healthy organs/tissues, while ACE2 was only highly expressed in the ileum. ACE2 had low expression and showed no significant difference between healthy control and COVID-19 patient samples across 7 organs/tissues. On the other hand, BSG had high expression in both healthy controls and COVID-19 patients across airway, nasal mucosa, and PBMC samples. We also analyzed the binding affinity between receptors and variant spikes (Fig. 1C) and found that BSG had the highest predicted binding affinity with 5 out of 7 strains.

Similar to previous findings [61], we observed the highest expression of BSG in SARS-CoV-2-RNA-positive cells when analysing COVID-19 patient single-cell datasets. Moderate correlations were also observed between the expression levels of BSG in macrophages, plasma cells, and T/NK cells and the abundance of viral RNA (Fig. 8F). BSG has been found to be expressed in all cell types, including immune cells [62]. Some studies support BSG as a novel pathway for SARS-CoV-2 entry into human cells [16], [28], [63], indicating its significance in COVID-19 infection. However, it is important to note that we cannot determine whether the high levels of receptors in cells are pre-existing or a result of infection. It is possible that cells and tissues with higher innate receptor expression are more susceptible to viral attack, or that viral entry induces host cells to express more receptors, thereby exacerbating infection. This may help explain the differential risk of infection progression in certain populations or organ systems. Additionally, our findings indicate that the BSG receptor plays a crucial role in multi-organ injuries caused by COVID-19. We observed high expression levels of BSG in the heart and testis (Fig. 2A), which aligns with previous studies reporting heart damage and reproductive issues in COVID-19 patients [64]. Our single-cell atlases suggest that the susceptibility of cells involved in spermatogenesis stages may contribute to azoospermia in COVID-19 patients [65].

These pieces of evidence strongly suggest that BSG is involved in SARS-CoV-2 infection across various human organs, leading to multi-organ injuries and long-COVID symptoms. Long-COVID refers to the persistence of symptoms even after the acute infection is cleared. The US Centers for Disease Control and Prevention (CDC) describes sequelae more than four weeks after initial infection as long-COVID [66], and it has been observed that SARS-CoV-2 can cause structural and functional damage to multiple organs. Long-term symptoms of COVID-19 have been reported in different tissues and organs [67], [68], [69]. While previous studies primarily focused on the ACE2 receptor as the binding site for SARS-CoV-2 [70], [71], our research highlights the widespread expression of the BSG receptor in multiple organs, suggesting its role in the diverse symptoms of COVID-19. Furthermore, clinical statistics indicate that long-COVID symptoms are more prevalent in adults and the elderly (UK Office for National Statistics. Prevalence of long-COVID symptoms and COVID-19 complications. 2020), which aligns with our finding of high BSG expression in this group. Anti-BSG antibodies and BSG inhibitors, such as meplazeumab [72]and pseudolaric acid B [73], are already available for treating various diseases. These antibodies can competitively bind to BSG, while inhibitors can reduce BSG abundance, thereby decreasing the chances of SARS-CoV-2 invading host cells. Repurposing or improving these existing drugs could be a viable option for addressing emerging SARS-CoV-2 variant strains.

### 3.3 Special expression patterns of ASGR1 imply the potential attack in liver

Chai's clinical study on healthy livers showed that ACE2 expression in liver cells is very low, but the liver can still be attacked by SARS-CoV-2 [74]. Qiao et al. [60] also found abnormal liver function in COVID-19 patients, especially in critically ill patients, suggesting that hepatocytes can be targeted by the virus. Additionally, ASGR1, another important receptor, is highly expressed in the liver (Figs. 2A, 3C, 4 and 6). Considering the reports [75] of multiple organ injuries in COVID-19 patients, it is suggested that ASGR1 may be a key receptor for SARS-CoV-2 in liver attacks. Recently, there have been 1010 cases of acute hepatitis in children of unknown cause reported in 35 countries, with 22 deaths by July 8, 2022 (www.who.int). Adenovirus infection has been associated with this hepatitis case [76]. Adenovirus is a common pathogen known to cause severe liver infections in immunocompromised populations, such as children [77]. Previous studies have shown that a high level of ASGR1 inhibits migration and invasion of hepatocellular carcinoma (HCC) cells [78], which may be related to immune activities in liver diseases and infections.

This study found that ASGR1 expression in children is significantly lower compared to adults and the elderly (Fig. 2B). The low level of ASGR1 in children may be related to immune dysfunction caused by adenovirus infection, which could explain the occurrence of acute hepatitis. It also suggests that the lower incidence of COVID-19 in children may be due to the potential role of ASGR1 as an important receptor for SARS-CoV-2. Conversely, the higher level of ASGR1 in adults and the elderly may be associated with fewer acute hepatitis infections but could pose a higher risk of COVID-19 infection. However, the exact roles of ASGR1 in COVID-19 and acute hepatitis in children are still unclear.

### 3.4 Receptor expression patterns in different ages explain the high prevalence of COVID-19 in elders

Our study found that the expression levels of six receptors were significantly lower in children compared to adults and elders (Fig. 2B). This is consistent with previous studies that have shown a lower incidence of COVID-19 in children and a higher risk in elders [79]. The expression level of the BSG receptor, which promotes SARS-CoV-2 entry into cells [80], also increases with age [81], providing more binding targets for the virus and contributing to higher infection rates in older individuals. Different racial populations also show variations in susceptibility to COVID-19. Africans have the highest susceptibility [82], [83], while Asians have a higher risk compared to Caucasians [84]. Within the same racial group, there are variations in susceptibility, as observed in the differences in hospitalization rates between South Asian and East Asian populations [85]. These variations may be attributed to genetic differences, such as ACE2 gene variants, which are more prevalent in East Asian populations and associated with resistance to the virus [86]. But in South Asian populations, the lower hospitalization rates observed may be related to frequent ACE2 mutations, which are believed to reduce the binding affinity with the novel coronavirus. Conversely, another ACE2 mutation that increases binding affinity with the spike protein is enriched in African populations [85]. These findings indicate that there are differences in receptor expression patterns among populations worldwide, which may help explain their varying susceptibility to COVID-19 infection.

### 3.5 Receptor expression patterns in patients with underlying diseases suggest different risks of COVID-19

Previous studies have shown that CVD and DM are important risk factors for the severity of COVID-19 infection [40]. Our research found that patients with CVD and DM have significantly higher expression of multiple SARS-CoV-2 receptors, particularly ACE2, at the transcriptomics level (Fig. 2C). ACE2 is a well-known receptor for SARS-CoV-2 and has been identified as a biomarker for CVD [87] and a therapeutic target for DM [88]. Additionally, the expression of ASGR1 was significantly increased in DM patients (Fig. 2C). These findings suggest that patients with CVD and DM may be more susceptible to severe COVID-19 infection due to multiple risk factors. Tumors have also been identified as a susceptibility risk factor for COVID-19 [89]. We observed a significant increase in the expression of ACE2 in lung cancer patients compared to controls (Fig. 2C), which is consistent with previous studies on lung adenocarcinoma [90]. However, the expressions of other receptors (AXL, BSG, KREMEN1, and TFRC) were significantly down-regulated in lung cancer patients (Fig. 2C). This implies that patients with lung cancer may have a relatively lower risk of contracting COVID-19 compared to those with CVD and DM.

Furthermore, we noticed that the expression of BSG is low in DM and lung cancer patients, in contrast to ACE2 (Fig. 2C). The binding of glycosylated ACE2 receptor to spike protein of cells depends on the cleavage of host transmembrane protease serine 2 (TMPRSS2) [91], and the role of BSG in Human Immunodeficiency Virus (HIV) and Severe Acute Respiratory Syndrome Coronavirus (SARS-CoV) infection is also closely related to its intracellular and transmembrane partners [15]. Besides this, BSG is also involved in HIV and SARS-CoV infection. Studies have shown that truncation of the cytoplasmic tail of BSG can prevent HIV infection [92]. Therefore, these molecules may influence the binding of SARS-CoV-2 to BSG and its entry into cells through a mechanism similar to TMPRSS2 to ACE2. ACE2 may compete with BSG in facilitating the entry of SARS-CoV-2 into cells. Meanwhile, ACE2 may be a major risk factor for COVID-19 infection in patients with DM and lung cancer.

### 3.6 Receptor expression patterns across primates indicate susceptibility similarities

Our transcriptomic analysis across primates suggested that heart, liver, kidney, lung, and testis of rhesus monkey, cynomolgus monkey, and chimpanzee may be susceptible to SARS-CoV-2. The gene expression patterns of the receptors of primates had strong similarity and indicated little difference in susceptibility across different primates. Previously reported laboratory experiments on animal infection with SARS-CoV-2 supported our findings. Vincent et al. detected SARS-CoV-2 RNA in lung, respiratory tract, lymph tissue, and gastrointestinal tract of infected rhesus monkey [93]. Wei et al. detected SARS-CoV-2 RNA in heart, stomach, colon, lung, and other tissues of infected rhesus monkey and cynomolgus monkey [94]. Salguero et al. found that SARS-CoV-2 was replicated in upper and lower respiratory tracts of rhesus monkey and cynomolgus monkey, leading to lung lesions and similar immune responses [95]. The transcriptomic expression patterns of the receptors across human and three primates indicate their strong similarities in molecular susceptibility.

## 4 Conclusions

In conclusion, we conducted systematic big data analysis on protein binding affinities, transcriptomic expressions, and single-cell atlases for the molecular susceptibility of different receptors to SARS-CoV-2 spike protein. Our study helps to explain the domination of delta and omicron variant strains, the high prevalence of COVID-19 in elders as well as the different risks for patients with underlying diseases. BSG may be involved in the SARS-CoV-2 infection processes across most human organs/tissues, and this might lead to multi-organ injuries and long COVID symptoms. Correlation analysis validated moderate associations between BSG and viral RNA abundance in multiple cell types. Importantly, similar patterns were observed in primates and validated in proteomic expressions. Although over four thousand experimental samples were involved in the source data, this study was purely based on public data analysis, which means the findings rely on the quality of public data and deserve for further wet lab validations by pharmaceutical industries. Overall, our study offers systematic insights into the molecular susceptibilities of different receptors to SARS-CoV-2 spike, and implicates important therapeutic targets for the development of receptor-specific vaccines and drugs for COVID-19.

## 5 Methods

The multi-omic analysis in this study included protein binding affinities between receptors and spike variants, transcriptomic analysis, single-cell atlases, and comparisons of different populations. The data sources for each analysis part are briefly summarized in Fig. S1.

### 5.1 Molecular docking and binding affinity computation

#### 5.1.1 Structure data acquisition and pre-processing

We performed the molecular docking simulation to predict the docking affinities between six receptors of human and the trimer spike protein of SARS-CoV-2. The variant strains of SARS-CoV-2 included the wild-type, alpha, beta, gamma, delta, lambda, and omicron strains. We obtained the protein structure data of the receptors and the spike protein either from the PDB database [38], the AlphaFold Protein Structure Database [96], or by homology modelling [97]. The structures of spike proteins from the wild-type, alpha, and beta variants are derived from crystallized proteins in the PDB, with all three monomers adopting an open conformation. The structures of spike proteins from other strains are generated through homology modelling, and similarly, only one monomer adopts an open conformation. The structure identifiers of spike protein in different SARS-CoV-2 strains are listed in Table 1. The receptor structures and their corresponding identifiers are listed in Table 2. Specifically, the structures of ACE2, ASGR1, KREMEN1, and TFRC receptors (PDB ID: 6M1D, 1DV8, 5FWS, and 2NSU, respectively) were obtained from PDB. The structures of AXL and BSG receptors (ID: AF-P30530-F1 and AF-P35613-F1) were obtained from the AlphaFold Protein Structure Database. Notably, only ACE2 of these receptor structures was resolved simultaneously with the RBD of spike proteins. For the ACE2, ASGR1, KREMEN1, and TFRC receptors, we employed the pGenThreader [98] for fold recognition to ensure that the selected structures were the optimal choices (Table S1). For the BSG and AXL receptors, we used Chimera [99] to map the BSG and AXL receptor fragments from the PDB onto the 3D models obtained from AlphaFold to validate their accuracy (Fig. S3). The spike protein crystal structures of the strains of wild-type, alpha, and beta (PDB ID: 6VYB, 7N1X, and 7N1Q, respectively) were retrieved from PDB. The alpha and beta variants of the SARS-CoV-2 virus have intact spike proteins with the exception of partial residue deletions in the wild-type strain. Additionally, both variants have a single monomeric RBD in an open conformation. For the remaining four variants, corresponding spike protein crystal structures cannot be directly obtained from the PDB. Therefore, we chose to use the SWISS-MODEL 3D homology modelling tool [100] to model the spike protein structures of the gamma, delta, lambda, and omicron strains. Specifically, we used the amino acid sequences of the variants as input sequences for template searching in homology modelling. The templates were selected based on GMQE, QSQE, and Identity scores in descending order, and the top-ranked open conformation crystal structures (PDB ID: 7KRR.1.A and 7N1V.1.A) were chosen as templates for homology modelling. Before processing the molecular docking simulation, the crystal structures of spike protein and receptor proteins were pre-processed by PyMOL (Version 2.4.1) to remove ligands and water molecules that do not belong to the protein itself, and add hydrogen atoms.

#### 5.1.2 Docking simulation between the complete structure of spike protein and its receptors

The docking software HADDOCK [101] was utilized to dock six receptors with the trimer spike protein of seven SARS-CoV-2 strains (Table S2). Actually, several software tools of molecular docking simulation are available, including HADDOCK, SwarmDock [102], ZDOCK [103], BIPSPI [104], FRODOCK [105], and GalaxyTonyDock_A [106] (Table S3). ZDOCK, BOIPSPI, and FRODOCK do not allow to assign docking residues and have input limit of molecular size; GalaxyToingDock_A also has input limit of molecular size; and SwarmDock takes too long (1–2 weeks) to complete a simulation job on its server. Therefore, we finally chose the prevalent tool HADDOCK, which allows residue assignation and has relatively short run time without input limit of molecular size.

We employed the HADDOCK software (version 2.2) to perform molecular docking simulations with receptors using a fixed-point docking method [101]. The docking residues assigned for the simulations are provided in Table S2. Based on in vitro binding experiments, we performed a screening of extracellular receptor binding sites. These selected sites were designated as the "active site" parameters of the receptor, as they are more likely to be the initial points of interaction with spike proteins. For the binding sites of spike proteins, we designated the RBD or NTD regions as the "active site" parameters. Research has confirmed that these six receptors exhibit varying degrees of binding to these regions [31], [91], [107]. The default values were retained for other parameters.

Subsequently, the protein binding affinities in terms of binding free energy were calculated for the top four conformations obtained from the docking results using the HawkDock Server [108]. HawkDock, based on the MM/GBSA algorithm, has demonstrated superior performance in predicting relative binding free energies [109] and is widely utilized for protein binding affinity calculations [110], [111] and complex model predictions [112], [113]. The binding affinities of the top four conformations for each variant receptor pair are shown in Table S4.

### 5.2 Transcriptomic and proteomic analysis at bulk-RNA-seq level

We analysed the expression levels of these six receptors in multiple organs of human and primate at the transcriptomic level using publicly available datasets. Furthermore, we explored the impact of age and underlying diseases on the distribution of these receptors. Protein-level analysis provided additional validation in our study.

#### 5.2.1 Data collection

We firstly collected public omics data of healthy samples, including transcriptomic expression profiles of 2386 human samples (2312 form GTEx and 74 from GEO) and 319 primate samples (*Macaca mulatta, Macaca fascicularis,* and *Pan troglodytes*). The human transcriptomic data at tissue level were downloaded from the GTEx database [114]. The transcriptomic data of the three primates contained 27 GSE datasets from the GEO database [115]. The data contained the genome-wide expression profiles of healthy individuals of each species. The data types included the count matrices and the expression matrices of FPKM (fragments per kilobase million), RPKM (reads per kilobase million), TPM (transcripts per million) and CPM (counts per million) [116]. The Ensembl gene identifiers of the receptors from the primates are listed in Table S5. The median expression matrix of TPM values for human genes was used to generate the median expression profile. The above two datasets contained the samples of 980 individuals (Version V8, 2017–06–05). The publicly data sets used in this study are detailed in Table S6.

#### 5.2.2 Transcriptomic data analysis at bulk-RNA-seq level

To construct the expression profiles of the receptors at the transcriptomics level, we used the median expression as the representative value for the expression distribution of a gene in a certain tissue. The median expression avoided the issue that the average expression was susceptible to extreme values. We used the bubble diagrams to display the median expression profiles of the receptors in different tissues.

In our collected data, all human samples are in CPM format, so no changes were made to them. The animal samples, on the other hand, include CPM, TPM, RPKM, FPKM, and raw counts. To standardize the data types, we performed data conversion and heatmap visualization on the expression profile datasets of three primate species. We performed data conversion and heatmap visualization on the expression profile datasets of three primates. TPM and CPM normalizations were firstly conducted to summarize the count matrix, the FPKM and RPKM expression matrices, thereby to compare the expression profiles across primates and tissues. In this step, we converted the count matrix to the CPM value matrix (Eq. 1), and transformed the FPKM and RPKM values to the TPM value matrix ((2), (3)). The original expression spectrums of TPM and CPM values were not converted. We then used the centroid clustering method (pheatmap in R) [51] to illustrate the TPM and CPM expression patterns of three primates.

(1) $$\begin{array}{c} {\mathrm{CPM}}_{i}=\left(\frac{X_{i}}{\sum_{j}X_{j}}\right)*10^{6}\# \end{array}$$

(2) $$\begin{array}{c} {\mathrm{TPM}}_{i}=\left(\frac{{\mathrm{RPKM}}_{i}}{\sum_{j}{\mathrm{RPKM}}_{j}}\right)*10^{6}\# \end{array}$$

(3) $$\begin{array}{c} {\mathrm{TPM}}_{i}=\left(\frac{{\mathrm{FPKM}}_{i}}{\sum_{j}{\mathrm{FPKM}}_{j}}\right)*10^{6}\# \end{array}$$

In the above equations, ${\mathrm{TPM}}_{i}$ and ${\mathrm{CPM}}_{i}$ are the TMP expression value and the CPM expression value for a given gene respectively; $X_{i}$ is the counts of a given gene in the count matrix; $\sum_{j}X_{j}$ is the sum of all genes in the count matrix; ${\mathrm{RPKM}}_{i}$ is the RPKM value for a give gene in the RPKM expression profile; $\sum_{j}{\mathrm{RPKM}}_{j}$ is the sum of RPKM values for all genes in the RPKM expression profile; ${\mathrm{FPKM}}_{i}$ is the FPKM value for a given gene in the FPKM expression profile; and $\sum_{j}{\mathrm{FPKM}}_{j}$ is the sum of FPKM values for all genes in the FPKM expression profile. It is worth noting that in the comparative analysis of primate data, we ultimately standardized all the data to CPM and TPM formats, and compared them separately.

### 5.3 Expression comparison among different age groups

The RNA-seq datasets of 939 whole blood samples from children (1–19 years), adults (20–59 years), and elders (60–79 years) were used for differential gene expression analysis. In addition, the expression profiles of healthy children groups came from GSE163634 in Gene Expression Omnibus database (GEO), adult and elder samples from GTEx. In order to unify standards, all expression matrices were normalized from FPKM to TPM and scaled by $\log_{2}\left(\mathrm{TPM}+1\right)$. Wilcoxon rank sum test was finally conducted to pairwise compare the distribution of receptor expression values at these three groups. The significance threshold *P*-value was 0.05.

### 5.4 Expression comparison among different underlying disease states

The RNA-seq datasets of cardiovascular disease (CVD) and diabetes mellitus (DM) from Gene Expression Omnibus database (GEO) as well as the TCGA database of lung cancer were used for the expression difference analysis. The numbers of patient and healthy samples are listed in Table 3. In addition, the expression profiles of control groups were healthy heart, pancreas, and lung samples from GTEx. In order to unify standards, all expression matrices were normalized by TPM and scaled by$\;\log_{2}\left(\mathrm{TPM}+1\right)$. Then, the Wilcoxon test was performed for ACE2, ASGR1, AXL, BSG, KEEMEN1, and TFRC according to the expression of each gene between disease group and healthy group. The significance threshold *P*-value was 0.05. Due to the involvement of multiple database sources in the bulk-RNA transcriptome of only the heart and pancreas healthy samples, we selected six genes as references. These include two housekeeping genes (FDX1 and UBE2K) [117], [118] and four genes (TNNT2 and ACTC1 for the heart [119], [120], EIF2S1 and GJA1 for the pancreas [121], [122]) that play foundational roles in their respective organs. By comparing the expression levels of these genes in healthy samples from different database sources (Fig. S4), we demonstrated the comparability of bulk-RNA transcripts obtained from different databases.

**Table 3:** The numbers of patient and healthy samples from the collected RNA-seq datasets for the expression comparison among different underlying disease states.

**Dataset**	**Patient samples**	**Normal samples**	**Total samples**
GSE48166	15 ICM	0	15
GSE57311	2 DCM; 1 ICM	0	3
GSE46224	8 ICM; 8 NICM	0	16
GSE89021	10 TID	0	10
GSE41762	20 T2D	0	20
TCGA	1129 lung cancer	0	1129
GTEx heart	0	861	861
GTEx pancreas	0	328	328
GTEx lung	0	578	578

### 5.5 Transcriptomic data analysis at single-cell level

The single cell transcriptomic data of 163 samples for healthy people and 90 samples for COVID-19 patients in total were collected from the UCSC Cell Browser [43]. The samples contained more than 1.2 million cells from liver, testis, nasal mucosa, prostate, pancreas, lung, ovary, colon, rectum, etc. In addition, 222,960 cells from a total of 23 COVID-19 patients in GSE145926 and GSE155249 from the GEO database were used to investigate correlations between virus infection and the presence of specific receptors. The expression matrix file, the cell type annotation file, and the corresponding cell coordination file with dimension reduction were used to plot the t-SNE and UMAP cell cluster diagrams and the expression diagrams.

The count matrixes of the single cells were loaded into a Seurat object using Seurat (V4.0.5) in R package [123], where further filtering was done to remove cells with fewer than 200 genes expressed or more than 15% of mitochondrial gene percentage were removed. Then an upper cut-off for the number of unique molecular identifiers (UMIs) was manually determined for each sample based on gene count versus UMI count, with values ranging between 50,000 and 200,000 UMIs. After quality control, we applied the “NormalizeData” function to normalize the expression matrices with default parameters, which included the use of the “LogNormalize” method and “scale.factor= 10000”. The top 2000 highly variable genes were identified by the “vst” method implemented in the “FindVariableFeatures” function for downstream analysis.

For the single-cell datasets from healthy and patients, the “SelectIntegrationFeatures” function was applied to select 2000 genes to integrate multiple datasets. To remove batch effects, the “RPCA” method wrapped in the “FindIntegrationAnchors” function was applied to find the anchor genes, and subsequently we used the “IntegrateData” function to integrate multiple datasets into an integrated dataset. The integrated dataset was scaled by the “ScaleData” function and the first 50 principle components (PCs) were calculated by “RunPCA” function. The top 50 PCs were adopted to automatic cluster using the “FindNeighbors” and “FindClusters” functions. The resolution parameter was set as 0.8 to obtain finer results. Based on the top 50 PCs, we performed UMAP and t-SNE to generated scatter plot in two-dimension space. “FeaturePlot” was used to visualize expression level of receptor genes.

### 5.6 Proteomic expression profiling for validation

To verify the results of human transcriptomic expression at proteomics level, we searched the 6 receptor proteins and retrieved the expression patterns of at human tissue level from the website of Proteomics DB database (https://www.proteomicsdb.org/) [124]. The protein expression of each receptor gene in different parts of human body was displayed in the expression graphs of human body using the median of standardized iBAQ intensity values [125].

## Ethics approval and consent to participate

Not applicable.

## Funding

This work was supported by the grants of National Key R&D Program of China (2021YFF1200900 and 2021YFF1200903), Guangdong Basic and Applied Basic Research Foundation (2022B1515120077), 10.13039/501100003453Natural Science Foundation of Guangdong Province (2021A1515012108), and Support Scheme of Guangzhou for Leading Talents in Innovation and Entrepreneurship (2020007).

## CRediT authorship contribution statement

**Weizhong Li:** Conceptualization, Supervision, Resources, Funding acquisition, Writing - original draft, Writing - review & editing. **Fanjie Wu:** Methodology, Formal analysis, Investigation, visualization, Writing - original draft. **Chenghao Lin:** Methodology, Formal analysis, Investigation, visualization; **Yuetong Han and Dingli Zhou:** Data curation, Validation. **Kang Chen and Minglei Yang:** Investigation; **Qingyuan Xiao:** Data curation. **Haiyue Zhang:** Project administration, Writing - review & editing.

## Declaration of generative AI and AI-assisted technologies in the writing process

During the preparation of this work the authors used ChatGPT in order to proofread the manuscript. After using this tool, the authors reviewed and edited the content as needed and take full responsibility for the content of the publication.

## Declaration of Competing Interest

The authors declare that they have no known competing financial interests or personal relationships that could have appeared to influence the work reported in this paper.
