# Supplementary material for: Multi-omic analysis characterizes molecular susceptibility of receptors to SARS-CoV-2 spike protein
Source: Comput Struct Biotechnol J. 2023 Nov 10;21:5583–600. doi: 10.1016/j.csbj.2023.11.012 (PMC10681948; doi:10.1016/j.csbj.2023.11.012)
Supplement: Supplementary file 1 — Supplementary material [file mmc1.docx]

Supplementary Figures


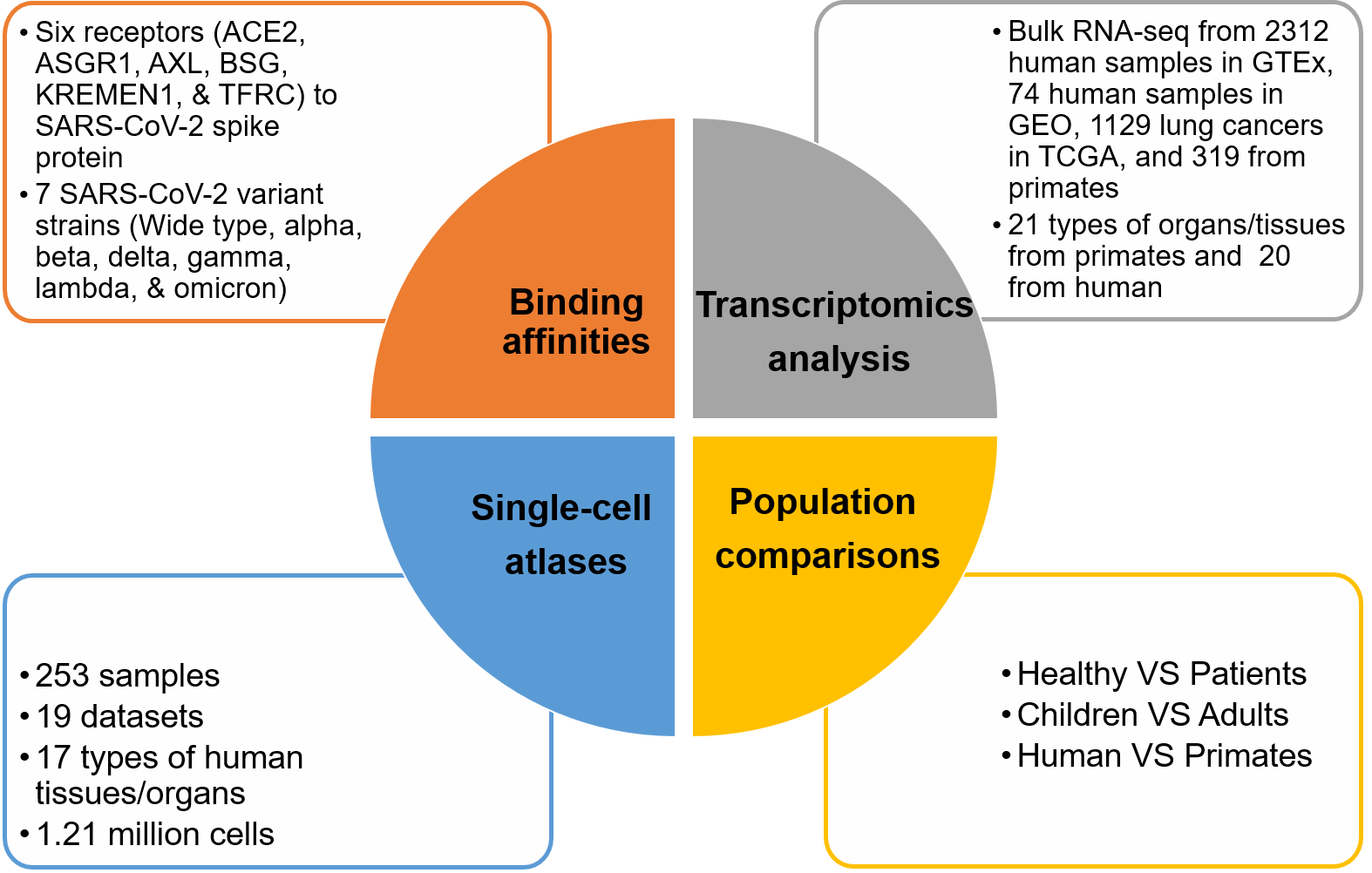


Fig. S1.

The data sources for each analysis part in this study. The analyses included binding affinities between receptors and spike protein variants, transcriptomics analysis, single-cell atlases, and comparisons of different populations.


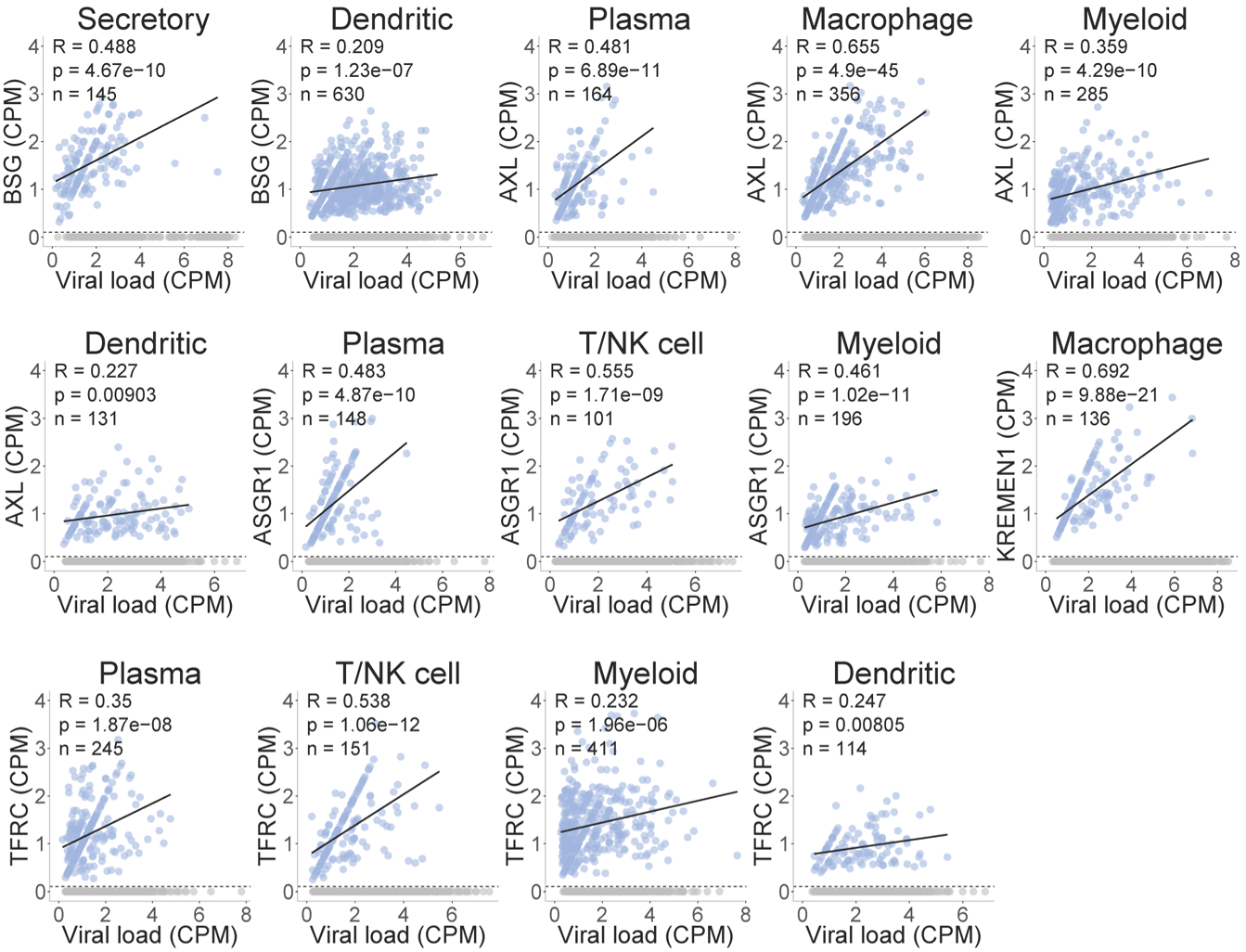


Fig. S2.

Pearson’s correlations of specific receptors’ expression patterns in multiple cell types (n>100, p<0.05) with viral load (CPM) (zero-expression cells were excluded from regression analysis to reduce the effects of dropouts).


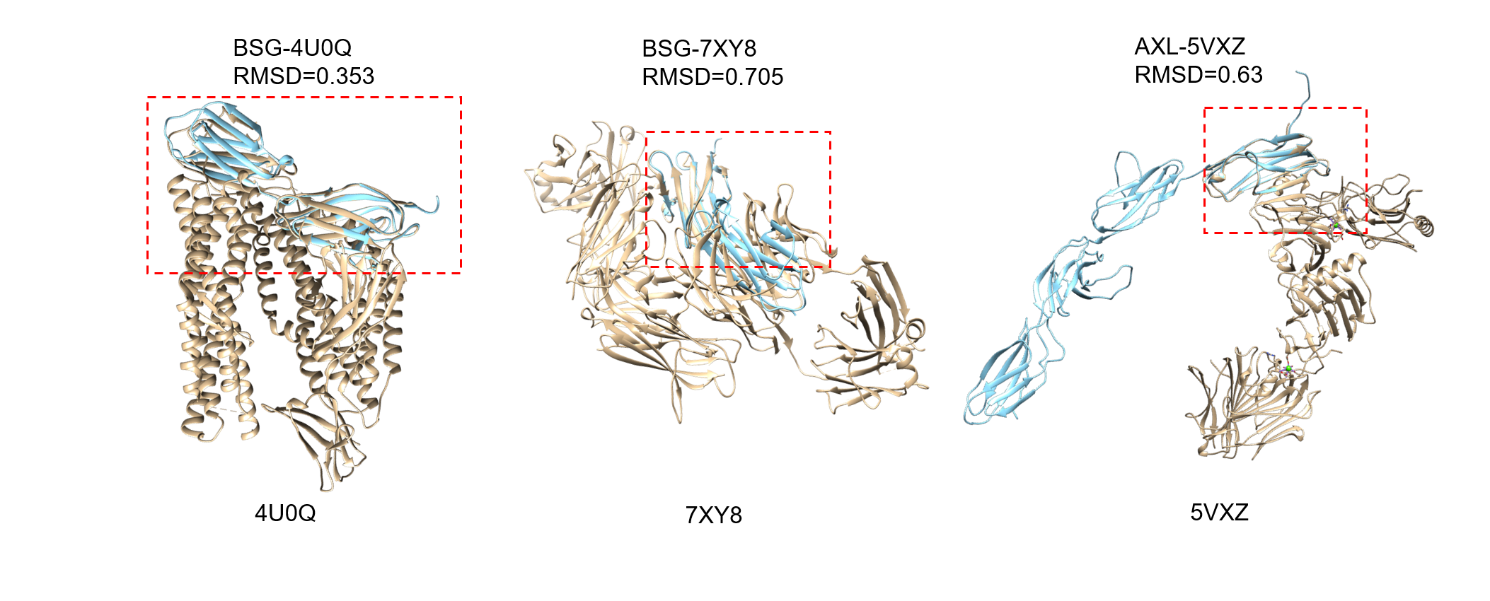


Fig. S3.

The crystal structure fragments of BSG (4U0Q and 7XY8) and AXL (5VXZ) were mapped onto the 3D models of BSG and AXL derived from AlphaFold.


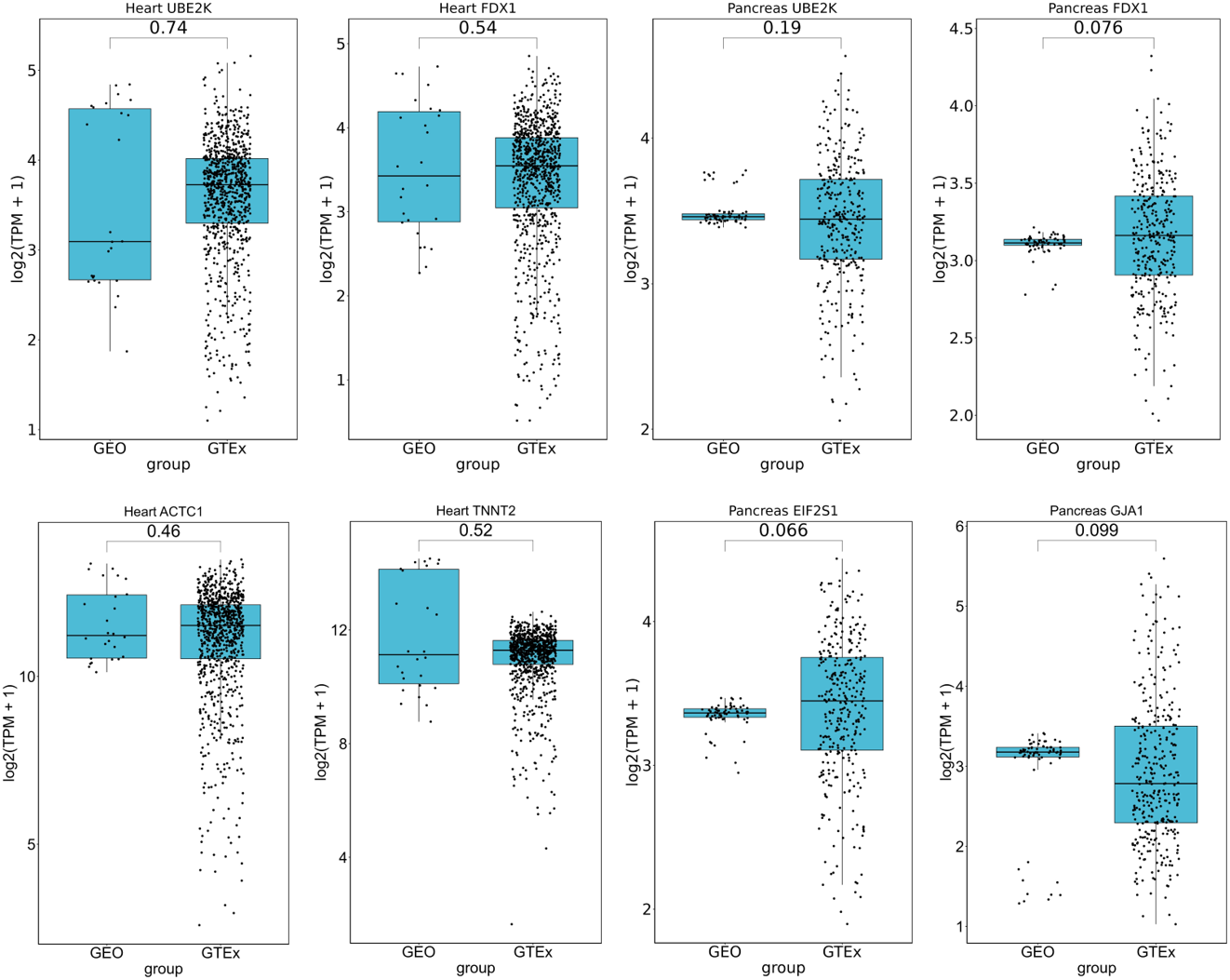


Fig. S4.

Transcriptomic expression comparisons of the reference genes in healthy samples of the heart and pancreas from different database sources. Housekeeping gene: FDX1 and UBE2K; Key gene of heart: TNNT2 and ACTC1; Key gene of pancreas: EIF2S1 and GJA1.

Supplementary Tables

**Table S1. ACE2, ASGR1, KREMEN1 and TFRC structurally related proteins sampled by pGenThreader**

| **Receptor template** | **Net Score** | **Aln Score** | **Aln Length** | **Target Len** | **Fold** | **PDB_ID** | **PDB title** | **Structure** | **Organism** |
| --- | --- | --- | --- | --- | --- | --- | --- | --- | --- |
| ACE2 (6m1d) | / | / | / | / | / | 6m1d | Ace2-b0at1 complex, open conformation | Sodium-dependent neutral amino acid transporter b(0)at1 | Homo sapiens |
| ACE2 (6m1d) | 331.831 | 1958.2 | 597 | 597 | 1r42A0 | 1r42 | Native human angiotensin converting enzyme-related carboxypeptidase (ace2) | Angiotensin i converting enzyme 2 | Homo sapiens |
| ACE2 (6m1d) | 325.963 | 1930.4 | 581 | 586 | 6h5wA0 | 6h5w | Crystal structure of human angiotensin-1 converting enzymE C-domain in complex with omapatrilat | Angiotensin-converting enzyme | Homo sapiens |
| ACE2 (6m1d) | 319.991 | 1894.1 | 594 | 607 | 5ambA0 | 5amb | Crystal structure of the angiotensin-1 converting enzyme n-domain in complex with amyloid-beta 35-42 | Angiotensin-converting enzyme | Homo sapiens |
| ACE2 (6m1d) | 118.295 | 590.0 | 521 | 654 | 2o36A0 | 2o36 | Crystal structure of engineered thimet oligopeptidase with neurolysin specificity in neurotensin cleavage site | Thimet oligopeptidase | Homo sapiens |
| ASGR1 (1dv8) | / | / | / | / | / | 1dv8 | Crystal structure of the carbohydrate recognition domain of the h1 subunit of the asialoglycoprotein receptor | Asialoglycoprotein receptor 1 | Homo sapiens |
| ASGR1 (1dv8) | 118.897 | 711.8 | 128 | 130 | 5jq1A0 | 5jq1 | Efficient targeting of the asialoglycoprotein receptor by polyvalent display of a compact galactosamine mimic | Asialoglycoprotein receptor 1 | Homo sapiens |
| ASGR1 (1dv8) | 83.448 | 476.0 | 121 | 147 | 4zesA0 | 4zes | Blood dendritic cell antigen 2 (bdca-2) complexed with methyl- mannoside | C-type lectin domain family 4 member c | Homo sapiens |
| ASGR1 (1dv8) | 83.093 | 465.0 | 121 | 130 | 5b1xA0 | 5b1x | Crystal structure of human dendritic cell inhibitory receptor (dcir) c-type lectin domain in complex with biantennary glycan | C-type lectin domain family 4 member a | Homo sapiens |
| ASGR1 (1dv8) | 79.764 | 443.0 | 121 | 143 | 5vybA0 | 5vyb | Structure of the carbohydrate recognition domain of dectin-2 complexed with a mammalian-type high mannose man9glcnac2 oligosaccharide | C-type lectin domain family 6 member a | Homo sapiens |
| KREMEN1 (5fws) | / | / | / | / | / | 5fws | Wnt modulator kremen crystal form i at 1.90a | Kremen protein 1 | Homo sapiens |
| KREMEN1 (5fws) | 131.240 | 714.4 | 262 | 288 | 5fwuA0 | 5fwu | Wnt modulator kremen crystal form ii at 2.8a | Kremen protein 1 | Homo sapiens |
| KREMEN1 (5fws) | 78.155 | 369.0 | 351 | 756 | 4durA0 | 4dur | The x-ray crystal structure of full-length type ii human plasminogen | Plasminogen | Homo sapiens |
| KREMEN1 (5fws) | 75.786 | 355.0 | 248 | 457 | 3kq4B0 | 3kq4 | Structure of intrinsic factor-cobalamin bound to its receptor cubilin | Gastric intrinsic factor | Homo sapiens |
| KREMEN1 (5fws) | 71.388 | 335.0 | 113 | 119 | 2wnoA0 | 2wno | X-ray structure of cub_c domain from tsg-6 | Tumor necrosis factor-inducible gene 6 protein | Homo sapiens |
| KREMEN1 (5fws) | 67.146 | 302.0 | 152 | 431 | 2qqoA0 | 2qqo | Crystal structure of the a2b1b2 domains from human neuropilin-2 | Neuropilin-2 | Homo sapiens |
| KREMEN1 (5fws) | 66.444 | 302.0 | 157 | 436 | 2qqmA0 | 2qqm | Crystal structure of the a2b1b2 domains from human neuropilin-1 | Neuropilin-1 | Homo sapiens |
| KREMEN1 (5fws) | 66.208 | 290.0 | 163 | 267 | 3demA0 | 3dem | Cub1-egf-cub2 domain of human masp-1/3 | Complement factor masp-3 | Homo sapiens |
| KREMEN1 (5fws) | 63.690 | 336.0 | 182 | 253 | 1ki0A0 | 1ki0 | The x-ray structure of human angiostatin | Angiostatin | Homo sapiens |
| TFRC (2nsu) | / | / | / | / | / | 2nsu | Crystal structure of the ectodomain of human transferrin receptor fitted into a cryo-em reconstruction of canine parvovirus and feline transferrin receptor complex | Transferrin receptor protein 1 | Homo sapiens |
| TFRC (2nsu) | 316.399 | 1763.8 | 632 | 633 | 3kasA0 | 3kas | Machupo virus gp1 bound to human transferrin receptor 1 | Transferrin receptor protein 1 | Homo sapiens |
| TFRC (2nsu) | 292.239 | 1635.0 | 619 | 690 | 3fedA0 | 3fed | The high resolution structure of human glutamate carboxypeptidase iii (gcpiii/naaladase ii) in complex with a transition state analog of glu-glu | Glutamate carboxypeptidase iii | Homo sapiens |
| TFRC (2nsu) | 289.360 | 1621.0 | 622 | 696 | 5o5tA0 | 5o5t | X-ray structure of human glutamate carboxypeptidase ii (gcpii) in complex with a urea based inhibitor psma 1007 | Glutamate carboxypeptidase 2 | Homo sapiens |
| TFRC (2nsu) | 276.466 | 1524.0 | 626 | 706 | 4tweA0 | 4twe | Structure of ligand-free n-acetylated-alpha-linked-acidic-dipeptidase like protein (naaladasel) | N-acetylated-alpha-linked acidic dipeptidase-like protein | Homo sapiens |
| TFRC (2nsu) | 67.531 | 277.0 | 270 | 323 | 2afwA0 | 2afw | Crystal structure of human glutaminyl cyclase in complex with n- acetylhistamine | Glutaminyl-peptide cyclotransferase | Homo sapiens |
| TFRC (2nsu) | 64.008 | 150.0 | 456 | 667 | 6iycA0 | 6iyc | Recognition of the amyloid precursor protein by human gamma-secretase | Nicastrin | Homo sapiens |
| TFRC (2nsu) | 63.855 | 245.0 | 269 | 313 | 3pb6X0 | 3pb6 | Crystal structure of the catalytic domain of human golgi-resident glutaminyl cyclase at PH 6.5 | Glutaminyl-peptide cyclotransferase-like protein | Homo sapiens |

**Table S2. Receptor active docking sites with spike proteins**

| **Receptor** | **Extracellular residue range of receptor** | **The surface docking sites of extracellular residues of receptor (manually curated based on in vitro binding experiments)** | **The docking regions of residues of spike protein** |
| --- | --- | --- | --- |
| ACE2 | 18-740 | 139, 281, 344, 406, 407, 432, 435, 436, 504, 527, 586, 587, 590, 638, 641,642, 645, 656, 660, 714, 728-740 | RBD (319-541) |
| ASGR1 | 62-291 | 153-162, 184, 185, 215, 216, 218,219, 220, 225, 226, 229-250, 253, 264, 269, 270, 278, 280 | NTD (27-303),  RBD (319-541) |
| AXL | 26-451 | 26-35, 66, 73, 74, 75, 77,78, 82, 90, 91, 123, 125, 138, 167, 168, 177, 178, 181, 184, 185, 186,187, 210, 230, 263-282, 302, 303, 304, 306, 323, 325, 327,335, 336, 338, 365, 368, 371, 372, 380, 381, 382, 386, 389, 390, 392, 393, 394, 396, 398, 400, 403, 413, 416, 419-424 | NTD (27-303) |
| BSG | 138-323 | 174, 185, 186, 187, 189, 231, 233, 236, 237, 243-249, 251, 252, 261, 262, 271, 309, 310, 313, 315, 317, 318, 320, 321, 322, 323 | RBD (319-541) |
| KREMEN1 | 21-392 | 43, 45, 46, 49-83, 88, 97, 103, 104, 105, 106, 108, 129, 140, 153, 154, 155, 173, 177, 217, 219, 237, 239,241, 250, 264, 265, 267, 275-287, 289, 290, 291, 292, 293, 295, 304, 307, 308 | NTD (27-303),  RBD (319-541) |
| TFRC | 89-760 | 122, 211, 291, 317, 318, 319, 320, 321, 322, 355, 469, 524, 525, 526, 527, 528, 665, 666, 736, 740, 744, 748, 753, 754, 756, 757, 758, 759, 760 | RBD (319-541) |

**Table S3. The software tools for molecular docking simulation.**

| **Software tool** | **Residue assignation for docking** | **Input limit of molecular size** | **Run time** |
| --- | --- | --- | --- |
| HADDOCK | Yes | No | Short (1-2 days) |
| SwarmDock | Yes | No | Long (1-2 weeks) |
| ZDOCK | No | Yes | Short |
| BIPSPI | No | Yes | Short |
| FRODOCK | No | Yes | Short |
| GalaxyTongDock_A | Yes | Yes | Short |

**Table S4.** The binding affinities of top four conformations by HawkDock for each variant-receptor pair.

| **Variant** | **Receptor (or average)** | **Conformation 1** | **Conformation 2** | **Conformation 3** | **Conformation 4** | **Average** |
| --- | --- | --- | --- | --- | --- | --- |
| Wild type | ACE2 | -75.94 | -94.53 | -84.04 | -54.2 | -77.1775 |
| Wild type | ASGR1 | -88.43 | -122.35 | -111.01 | -93.86 | -103.9125 |
| Wild type | AXL | -126.03 | -105.07 | -105.05 | -143.61 | -119.94 |
| Wild type | BSG | -146.33 | -133.1 | -144.81 | -117.39 | -135.4075 |
| Wild type | KREMEN1 | -111.98 | -117.83 | -120.32 | -126.29 | -119.105 |
| Wild type | TFRC | -87.99 | -90.97 | -101.17 | -112.48 | -98.1525 |
| Wild type | Average | -108.9491667 | -108.9491667 | -108.9491667 | -108.9491667 | -108.9491667 |
| Alpha | ACE2 | -135.4 | -134.08 | -122.68 | -109.07 | -125.3075 |
| Alpha | ASGR1 | -96.17 | -111.37 | -84.77 | -90.84 | -95.7875 |
| Alpha | AXL | -112.08 | -125.6 | -83.64 | -135.94 | -114.315 |
| Alpha | BSG | -123.9 | -173.28 | -131.74 | -137.12 | -141.51 |
| Alpha | KREMEN1 | -137.67 | -91.82 | -75.68 | -65.68 | -92.7125 |
| Alpha | TFRC | -71.89 | -141.19 | -142.17 | -135.42 | -122.6675 |
| Alpha | Average | -115.3833333 | -115.3833333 | -115.3833333 | -115.3833333 | -115.3833333 |
| Beta | ACE2 | -147.42 | -150.82 | -145.73 | -142.29 | -146.565 |
| Beta | ASGR1 | -139.86 | -115.65 | -111.51 | -140.85 | -126.9675 |
| Beta | AXL | -104.69 | -151.35 | -117.79 | -135.91 | -127.435 |
| Beta | BSG | -104.33 | -118.41 | -139.43 | -108.72 | -117.7225 |
| Beta | KREMEN1 | -157.93 | -125.64 | -117.97 | -135.11 | -134.1625 |
| Beta | TFRC | -121.44 | -174.68 | -138.41 | -136.59 | -142.78 |
| Beta | Average | -132.6054167 | -132.6054167 | -132.6054167 | -132.6054167 | -132.6054167 |
| Delta | ACE2 | -139.42 | -153.46 | -119.76 | -131.88 | -136.13 |
| Delta | ASGR1 | -136.35 | -142.9 | -152.78 | -109.98 | -135.5025 |
| Delta | AXL | -114.93 | -133.9 | -124.19 | -121.16 | -123.545 |
| Delta | BSG | -149.43 | -134.14 | -136.59 | -151.04 | -142.8 |
| Delta | KREMEN1 | -131.01 | -146.04 | -147.69 | -123.66 | -137.1 |
| Delta | TFRC | -147.01 | -111.58 | -140.74 | -131.52 | -132.7125 |
| Delta | Average | -134.6316667 | -134.6316667 | -134.6316667 | -134.6316667 | -134.6316667 |
| Gamma | ACE2 | -131.06 | -122.93 | -139.89 | -82.82 | -119.175 |
| Gamma | ASGR1 | -121.74 | -118.64 | -133.82 | -130.96 | -126.29 |
| Gamma | AXL | -166.2 | -134.62 | -141.2 | -134.11 | -144.0325 |
| Gamma | BSG | -173.56 | -152.85 | -138.56 | -136.99 | -150.49 |
| Gamma | KREMEN1 | -103.23 | -108.12 | -103.17 | -88.9 | -100.855 |
| Gamma | TFRC | -93.96 | -81.91 | -132.3 | -115.21 | -105.845 |
| Gamma | Average | -124.4479167 | -124.4479167 | -124.4479167 | -124.4479167 | -124.4479167 |
| Lambda | ACE2 | -120.12 | -112.16 | -113.61 | -111.57 | -114.365 |
| Lambda | ASGR1 | -121.89 | -109.11 | -100.34 | -122.75 | -113.5225 |
| Lambda | AXL | -99.83 | -82.08 | -89.35 | -114.21 | -96.3675 |
| Lambda | BSG | -150.49 | -89.41 | -117.5 | -125.72 | -120.78 |
| Lambda | KREMEN1 | -140.3 | -137.14 | -144.44 | -119.54 | -135.355 |
| Lambda | TFRC | -126.01 | -158.32 | -125.29 | -97.22 | -126.71 |
| Lambda | Average | -117.85 | -117.85 | -117.85 | -117.85 | -117.85 |
| Omicron | ACE2 | -110.03 | -91.81 | -116.94 | -127.36 | -111.535 |
| Omicron | ASGR1 | -139.07 | -144.76 | -153.5 | -107.78 | -136.2775 |
| Omicron | AXL | -150.26 | -156.76 | -161.3 | -130.64 | -149.74 |
| Omicron | BSG | -183.87 | -140.92 | -170.99 | -129.23 | -156.2525 |
| Omicron | KREMEN1 | -141.83 | -159.28 | -128.96 | -110.27 | -135.085 |
| Omicron | TFRC | -135.18 | -131.8 | -114.59 | -136.49 | -129.515 |
| Omicron | Average | -136.4008333 | -136.4008333 | -136.4008333 | -136.4008333 | -136.4008333 |

**Table S5.** The Ensembl gene identifiers of 6 receptors of different primates.

| **Receptor** | **Human  (H*omo sapiens*)** | **Rhesus monkey  (*Macaca mulatta*)** | **Cynomolgus monkey (*Macaca fascicularis*)** | **Chimpanzee  (*Pan troglodytes*)** |
| --- | --- | --- | --- | --- |
| ACE2 | ENSG130234 | ENSMMUG14048 | ENSMFAG39194 | ENSPTRG21688 |
| ASGR1 | ENSG141505 | ENSMMUG3247 | ENSMFAG35586 | ENSPTRG8662 |
| AXL | ENSG167601 | ENSMMUG9145 | ENSMFAG39691 | ENSPTRG11018 |
| BSG | ENSG172270 | ENSMMUG18740 | ENSMFAG40111 | ENSPTRG10155 |
| KREMEN1 | ENSG183762 | ENSMMUG21370 | ENSMFAG35109 | ENSPTRG14214 |
| TFRC | ENSG72274 | ENSMMUG2114 | ENSMFAG711 | ENSPTRG15773 |

**Table S6. Publicly available data sets used in this study**

| **Dataset** | **Organism** | **Data type** | **Disease status** | **Sample** | **Release/Date** | **URL** |
| --- | --- | --- | --- | --- | --- | --- |
| GSE125483 | *Macaca fascicularis* | bulk RNA-seq | Healthy | 4 adiposes, 6 adrenals, 6 brains, 6 colons, 6 hearts, 6 livers, 6 lungs, 6 muscles, 6 pituitaries, 6 skins, 5 spleens, 6 thyroids, 8 testes | 15-Jul-19 | https://www.ncbi.nlm.nih.gov/geo/query/acc.cgi?acc=GSE125483 |
| GSE102830 | *Macaca fascicularis* | bulk RNA-seq | Healthy | 4 muscles, 4 brains, 4 thymuses, 4 livers, 4 hearts, 4 lungs, 4 kidneys | 26-Jul-18 | https://www.ncbi.nlm.nih.gov/geo/query/acc.cgi?acc=GSE102830 |
| GSE120935 | *Macaca fascicularis* | bulk RNA-seq | Healthy | 6 livers, 6 kidneys | 1-May-19 | https://www.ncbi.nlm.nih.gov/geo/query/acc.cgi?acc=GSE120935 |
| GSE140246 | *Macaca fascicularis* | bulk RNA-seq | Healthy | 6 brains | 31-Dec-19 | https://www.ncbi.nlm.nih.gov/geo/query/acc.cgi?acc=GSE140246 |
| GSE103825 | *Macaca fascicularis* | bulk RNA-seq | Healthy | 12 whole bloods | 12-Apr-18 | https://www.ncbi.nlm.nih.gov/geo/query/acc.cgi?acc=GSE103825 |
| GSE99463 | *Macaca fascicularis* | bulk RNA-seq | Healthy | 5 whole bloods | 9-Nov-17 | https://www.ncbi.nlm.nih.gov/geo/query/acc.cgi?acc=GSE99463 |
| GSE64538 | *Macaca fascicularis* | bulk RNA-seq | Healthy | 6 PBMCs | 29-Dec-14 | https://www.ncbi.nlm.nih.gov/geo/query/acc.cgi?acc=GSE64538 |
| GSE132998 | *Macaca fascicularis* | bulk RNA-seq | Healthy | 12 PBMCs | 20-Jun-19 | https://www.ncbi.nlm.nih.gov/geo/query/acc.cgi?acc=GSE132998 |
| GSE122785 | *Macaca fascicularis* | bulk RNA-seq | Healthy | 3 lungs | 28-Jun-19 | https://www.ncbi.nlm.nih.gov/geo/query/acc.cgi?acc=GSE122785 |
| GSE124002 | *Macaca fascicularis* | bulk RNA-seq | Healthy | 6 livers | 31-Dec-19 | https://www.ncbi.nlm.nih.gov/geo/query/acc.cgi?acc=GSE124002 |
| GSE130132 | *Macaca fascicularis* | bulk RNA-seq | Healthy | 12 brains | 26-Aug-19 | https://www.ncbi.nlm.nih.gov/geo/query/acc.cgi?acc=GSE130132 |
| GSE134706 | *Macaca fascicularis* | bulk RNA-seq | Healthy | 3 brains | 12-Dec-19 | https://www.ncbi.nlm.nih.gov/geo/query/acc.cgi?acc=GSE134706 |
| GSE137135 | *Macaca fascicularis* | bulk RNA-seq | Healthy | 15 lungs | 26-Nov-19 | https://www.ncbi.nlm.nih.gov/geo/query/acc.cgi?acc=GSE137135 |
| GSE104234 | *Macaca mulatta* | bulk RNA-seq | Healthy | 6 livers | 14-Mar-18 | https://www.ncbi.nlm.nih.gov/geo/query/acc.cgi?acc=GSE104234 |
| GSE56962 | *Macaca mulatta* | bulk RNA-seq | Healthy | 2 brains, 2 livers, 2 muscles | 1-Dec-19 | https://www.ncbi.nlm.nih.gov/geo/query/acc.cgi?acc=GSE56962 |
| GSE43520 | *Macaca mulatta* | bulk RNA-seq | Healthy | 1 brains, 1 testes | 19-Jan-14 | https://www.ncbi.nlm.nih.gov/geo/query/acc.cgi?acc=GSE43520 |
| GSE78165 | *Macaca mulatta* | bulk RNA-seq | Healthy | 12 retinas | 1-Jun-17 | https://www.ncbi.nlm.nih.gov/geo/query/acc.cgi?acc=GSE78165 |
| GSE49379 | *Macaca mulatta* | bulk RNA-seq | Healthy | 6 muscles, 6 kidneys | 30-May-14 | https://www.ncbi.nlm.nih.gov/geo/query/acc.cgi?acc=GSE49379 |
| GSE95736 | *Macaca mulatta* | bulk RNA-seq | Healthy | 1 ovaries, 3 testes, 4 livers | 7-Jun-17 | https://www.ncbi.nlm.nih.gov/geo/query/acc.cgi?acc=GSE95736 |
| GSE68507 | *Macaca mulatta* | bulk RNA-seq | Healthy | 4 testes | 7-May-16 | https://www.ncbi.nlm.nih.gov/geo/query/acc.cgi?acc=GSE68507 |
| GSE115785 | *Macaca mulatta* | bulk RNA-seq | Healthy | 12 bloods | 26-Aug-18 | https://www.ncbi.nlm.nih.gov/geo/query/acc.cgi?acc=GSE115785 |
| GSE124707 | *Macaca mulatta* | bulk RNA-seq | Healthy | 6 adiposes | 7-Jan-19 | https://www.ncbi.nlm.nih.gov/geo/query/acc.cgi?acc=GSE124707 |
| GSE56845 | *Macaca mulatta* | bulk RNA-seq | Healthy | 11 rectums | 17-Apr-14 | https://www.ncbi.nlm.nih.gov/geo/query/acc.cgi?acc=GSE56845 |
| GSE111077 | *Macaca mulatta* | bulk RNA-seq | Healthy | 15 rectums | 20-Sep-18 | https://www.ncbi.nlm.nih.gov/geo/query/acc.cgi?acc=GSE111077 |
| GSE97786 | *Macaca mulatta* | bulk RNA-seq | Healthy | 3 testes | 9-Oct-17 | https://www.ncbi.nlm.nih.gov/geo/query/acc.cgi?acc=GSE97786 |
| GSE50782 | *Pan troglodytes* | bulk RNA-seq | Healthy | 5 brains, 4 hearts, 4 kidneys, 5 livers, 5 testes | 1-Oct-13 | https://www.ncbi.nlm.nih.gov/geo/query/acc.cgi?acc=GSE50782 |
| GSE130871 | *Pan troglodytes* | bulk RNA-seq | Healthy | 2 brains | 20-Dec-19 | https://www.ncbi.nlm.nih.gov/geo/query/acc.cgi?acc=GSE130871 |
| GSE163634 | *Homo sapiens* | bulk RNA-seq | Healthy | 10 children bloods | 21-Dec-22 | <https://www.ncbi.nlm.nih.gov/geo/query/acc.cgi?acc=GSE163634> |
| GTEx project | *Homo sapiens* | bulk RNA-seq | Healthy | 24 aortas, 9 coronaries, 27 breasts, 12 colons, 18 esophaguses, 861 hearts, 3 kidneys, 5 livers, 578 lungs, 138 muscles, 6 ovaries, 328 pancreases, 9 prostates, 12 stomachs, 14 testes, 105 thyroids, 7 uteruses, 156 whole bloods | 24-Aug-15 | <https://www.ncbi.nlm.nih.gov/pmc/articles/PMC4547484/> |
| GSE48166 | *Homo sapiens* | bulk RNA-seq | Patient | 15 ICM hearts | 20-Jun-13 | <https://www.ncbi.nlm.nih.gov/geo/query/acc.cgi?acc=GSE48166> |
| GSE57344 | *Homo sapiens* | bulk RNA-seq | Patient | 2 DCM hearts, 1 ICM heart | 6-May-14 | <https://www.ncbi.nlm.nih.gov/geo/query/acc.cgi?acc=GSE57344> |
| GSE46224 | *Homo sapiens* | bulk RNA-seq | Patient | 8 ICM hearts, 8 NICM hearts | 19-Apr-13 | <https://www.ncbi.nlm.nih.gov/geo/query/acc.cgi?acc=GSE46224> |
| GSE89022 | *Homo sapiens* | bulk RNA-seq | Patient | 10 TID peripheral whole bloods | 21-Oct-16 | <https://www.ncbi.nlm.nih.gov/geo/query/acc.cgi?acc=GSE89022> |
| GSE41762 | *Homo sapiens* | bulk RNA-seq | Patient | 20 T2D islets | 22-Oct-12 | <https://www.ncbi.nlm.nih.gov/geo/query/acc.cgi?acc=GSE41762> |
| TCGA | *Homo sapiens* | bulk RNA-seq | Patient | 1129 lungs | 8-Sep-17 | [https://xenabrowser.net/datapages/?cohort= TCGA%20Lung%20Cancer%20(LUNG)&removeHub =https%3A%2F%2Fxena.treehouse.gi.ucsc.edu%3A443](https://xenabrowser.net/datapages/?cohort=TCGA%20Lung%20Cancer%20(LUNG)&removeHub=https%3A%2F%2Fxena.treehouse.gi.ucsc.edu%3A443) |
| GSE134809 | *Homo sapiens* | single cell RNA-seq | Healthy | 22 ileums, 5950 cells | 29-Aug-19 | <https://www.ncbi.nlm.nih.gov/geo/query/acc.cgi?acc=GSE134809> |
| GSE162864 | *Homo sapiens* | single cell RNA-seq | Healthy | 21 nasal mucosas, 25931 cells | 6-Apr-21 | <https://www.ncbi.nlm.nih.gov/geo/query/acc.cgi?acc=GSE162864> |
| ERP123138 | *Homo sapiens* | single cell RNA-seq | Healthy | 6 hearts, 387059 cells | 19-Aug-20 | <https://www.ebi.ac.uk/ena/browser/view/PRJEB39602?show=reads> |
| Figshare.11981034 | *Homo sapiens* | single cell RNA-seq | Healthy | 4 bronchiole epithelial cells, 14507 cells. 12 lungs, 39474 cells | 14-Mar-20 | [https://figshare.com/articles/dataset/Single-cell_RNA-Seq_of_human_primary_lung_and_bronchial_epithelium_cells /11981034/1](https://figshare.com/articles/dataset/Single-cell_RNA-Seq_of_human_primary_lung_and_bronchial_epithelium_cells/11981034/1) |
| Figshare.12436517 | *Homo sapiens* | single cell RNA-seq | Healthy | 5 nasals, 97734 cells | 21-Jul-20 | [https://figshare.com/articles/dataset /COVID-19_severity_correlates_with_airway_epithelium -immune_cell_interactions_identified_by_single-cell_analysis /12436517](https://figshare.com/articles/dataset/COVID-19_severity_correlates_with_airway_epithelium-immune_cell_interactions_identified_by_single-cell_analysis/12436517) |
| Figshare.14267219 | *Homo sapiens* | single cell RNA-seq | Healthy | 3 spleens, 7702 cells | 21-Jul-21 | [https://figshare.com/articles/dataset /Tabula_Sapiens_release_1_0/14267219](https://figshare.com/articles/dataset/Tabula_Sapiens_release_1_0/14267219) |
| Kidney Cell Atlas Project | *Homo sapiens* | single cell RNA-seq | Healthy | 14 kidneys, 33482 cells | 27-Sep-19 | <https://www.kidneycellatlas.org/> |
| GSE125970 | *Homo sapiens* | single cell RNA-seq | Healthy | 2 rectums, 3744 cells. 2 colons, 4275 cells. | 21-Oct-19 | <https://www.ncbi.nlm.nih.gov/geo/query/acc.cgi?acc=GSE125970> |
| GSE118127 | *Homo sapiens* | single cell RNA-seq | Healthy | 31 ovaries, 52513 cells | 13-Jun-19 | <https://www.ncbi.nlm.nih.gov/geo/query/acc.cgi?acc=GSE118127> |
| GSE84133 | *Homo sapiens* | single cell RNA-seq | Healthy | 4 pancreases, 7659 cells | 26-Sep-16 | <https://www.ncbi.nlm.nih.gov/geo/query/acc.cgi?acc=GSE84133> |
| [GSE117403](https://www.ncbi.nlm.nih.gov/geo/query/acc.cgi?acc=GSE117403) | *Homo sapiens* | single cell RNA-seq | Healthy | 16 prostates, 30023 cells | 4-Dec-18 | <https://www.ncbi.nlm.nih.gov/geo/query/acc.cgi?acc=GSE117403> |
| [GSE112013](https://www.ncbi.nlm.nih.gov/geo/query/acc.cgi?acc=GSE112013) | *Homo sapiens* | single cell RNA-seq | Healthy | 6 testes, 5286 cells | 15-Oct-18 | <https://www.ncbi.nlm.nih.gov/geo/query/acc.cgi?acc=GSE112013> |
| GSE115469 | *Homo sapiens* | single cell RNA-seq | Healthy | 5 livers, 8427 cells | 22-Oct-18 | <https://www.ncbi.nlm.nih.gov/geo/query/acc.cgi?acc=GSE115469> |
| GSE149689 | *Homo sapiens* | single cell RNA-seq | Healthy | 4 PBMC, 17512 cells | 6-Jul-20 | <https://www.ncbi.nlm.nih.gov/geo/query/acc.cgi?acc=GSE149689> |
| SCP1216 | *Homo sapiens* | single cell RNA-seq | Healthy | 3 hearts, 32395 cells | 25-Feb-21 | [https://singlecell.broadinstitute.org/single_cell /study/SCP1216/covid-19-heart-autopsy-samples](https://singlecell.broadinstitute.org/single_cell/study/SCP1216/covid-19-heart-autopsy-samples) |
| SCP1052 | *Homo sapiens* | single cell RNA-seq | Healthy | 3 lungs, 36677 cells | 25-Feb-21 | [https://singlecell.broadinstitute.org/single_cell /study/SCP1052/covid-19-lung-autopsy-samples](https://singlecell.broadinstitute.org/single_cell/study/SCP1052/covid-19-lung-autopsy-samples) |
| SCP1052 | *Homo sapiens* | single cell RNA-seq | COVID-19 Patient | 16 lungs, 79636 cells | 25-Feb-21 | [https://singlecell.broadinstitute.org/single_cell /study/SCP1052/covid-19-lung-autopsy-samples](https://singlecell.broadinstitute.org/single_cell/study/SCP1052/covid-19-lung-autopsy-samples) |
| SCP1213 | *Homo sapiens* | single cell RNA-seq | COVID-19 Patient | 15 livers, 46357 cells | 25-Feb-21 | [https://singlecell.broadinstitute.org/single_cell /study/SCP1213/covid-19-liver-autopsy-samples](https://singlecell.broadinstitute.org/single_cell/study/SCP1213/covid-19-liver-autopsy-samples) |
| SCP1214 | *Homo sapiens* | single cell RNA-seq | COVID-19 Patient | 11 kidneys, 33683 cells | 25-Feb-21 | [https://singlecell.broadinstitute.org/single_cell /study/SCP1214/covid-19-kidney-autopsy-samples](https://singlecell.broadinstitute.org/single_cell/study/SCP1214/covid-19-kidney-autopsy-samples) |
| SCP1216 | *Homo sapiens* | single cell RNA-seq | COVID-19 Patient | 15 hearts, 118750 cells | 25-Feb-21 | [https://singlecell.broadinstitute.org/single_cell /study/SCP1216/covid-19-heart-autopsy-samples](https://singlecell.broadinstitute.org/single_cell/study/SCP1216/covid-19-heart-autopsy-samples) |
| GSE166766 | *Homo sapiens* | single cell RNA-seq | COVID-19 Patient | 3 airways, 66668 cells | 28-Feb-21 | <https://www.ncbi.nlm.nih.gov/geo/query/acc.cgi?acc=GSE166766> |
| GSE149689 | *Homo sapiens* | single cell RNA-seq | COVID-19 Patient | 11 PBMC, 31270 cells | 6-Jul-20 | <https://www.ncbi.nlm.nih.gov/geo/query/acc.cgi?acc=GSE149689> |
| Figshare.12436517 | *Homo sapiens* | single cell RNA-seq | COVID-19 Patient | 19 nasals, 25931 cells | 21-Jul-20 | [https://figshare.com/articles/dataset /COVID-19_severity_correlates_with_airway_epithelium -immune_cell_interactions_identified_by_single-cell_analysis /12436517](https://figshare.com/articles/dataset/COVID-19_severity_correlates_with_airway_epithelium-immune_cell_interactions_identified_by_single-cell_analysis/12436517) |
| GSE145926 | *Homo sapiens* | single cell RNA-seq | COVID-19 Patient | 12 patients, 145283cells | 22-Apr-2020 | https://www.ncbi.nlm.nih.gov/geo/query/acc.cgi?acc=GSE145926 |
| GSE155249 | *Homo sapiens* | single cell RNA-seq | COVID-19 Patient | 11 patients, 77777cells | 05-Aug-2020 | https://www.ncbi.nlm.nih.gov/geo/query/acc.cgi?acc=GSE155249 |
